# Supplementary material for: A nitrogen-doped nanotube molecule with atom vacancy defects
Source: Nat Commun. 2020 Apr 14;11:1807. doi: 10.1038/s41467-020-15662-6 (PMC7156684; doi:10.1038/s41467-020-15662-6)
Supplement: Supplementary file 1 — Supplementary Information [file 41467_2020_15662_MOESM1_ESM.pdf]

Supplementary Information

A nitrogen-doped nanotube molecule with atom vacancy defects

Ikemoto *et al*.

Table of contents

Supplementary Methods 2
- Synthesis 2
- Locations of Defects 7
- Crystallography 9
- DFT Calculations 19
- Chromatograms 21
- NMR spectra 22

Supplementary References 37

Supplementary Methods

Synthesis

Flash silica gel column chromatography was performed on silica gel 60N (spherical and neutral gel, 40–50 μm, Kanto), and gel permeation chromatography (GPC) was performed on JAI LC-9104 systems equipped with a UV detector using JAI GEL 1H-40, 2H-40 and 2.5H-40 columns (eluent: $CHCl_3$). Analytical high-performance liquid chromatography (HPLC) for purity verifications was performed with two-types of columns (COSMOSIL πNAP, 4.6φ × 250 mm and COSMOSIL BuckyPrep, 4.6φ × 250 mm) in a column oven (40 °C) at the flow rate of 1.0 mL/min with a UV detection (280 nm). ^1H and ^{13}C NMR spectra were recorded on a JEOL RESONANCE JNM-ECA II 600 equipped with an UltraCOOL probe. Chemical shift values are given with respect to internal $CHCl_3$ for ^1H NMR (δ 7.26) and $CDCl_3$ for ^{13}C NMR (δ 77.16). Data are reported as follows: chemical shift, multiplicity (s = singlet, d = doublet, t = triplet), coupling constant in hertz (Hz) and a relative integration value. High-resolution mass spectra were performed on a Bruker Daltonics autoflex speed using matrix assisted laser desorption ionization (MALDI) method with dithranol as a matrix (ionization mode: reflector positive) or on a Bruker micrOTOF II spectrometer equipped with an APCI probe equipped with a DirectProbe (DIP). UV-vis spectroscopy was recorded on JASCO V670.

Anhydrous THF (stabilizer free), DMF and toluene were purified by a solvent purification system (GlassContour) equipped with columns of activated alumina and supported copper catalyst (Q-5)[1]. Milli Q water was used for reactions and degassed prior to use. All other chemicals were of reagent grade and used without any further purification. 3-chloro-5-*t*-butyl-phenylboronic acid pinacol ester was prepared according to the reported procedure[2]. The reactions were performed under N_2 atmosphere unless otherwise noted.

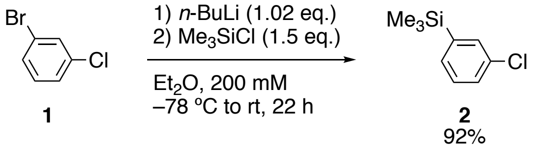

*1-Chloro-3-(trimethylsilyl)benzene (**2**)*: To a solution of 1-bromo-3-chlorobenzene (**1**) (23.0 g, 120 mmol) in diethyl ether (600 mL) was added a solution of *n*-butyllithium (122 mmol) in hexanes (78 mL) dropwise at –78 °C. After stirring for 15 min at –78 ºC, chlorotrimethylsilane (22.8 mL, 180 mmol) was added, and then the mixture was allowed to warm up to room temperature and stirred for 22 h. The reaction was quenched with water (300 mL). The mixture was extracted with chloroform (130 mL × 3), and the combined organic layer was dried over Na_2SO_4 and concentrated in vacuo. The crude material was purified by distillation (b.p. 65 °C at 3 Torr). The title compound was obtained as

a colorless oil in 92% yield (20.4 g, 110 mmol). Spectra of **2** were identical to data found in the literature[3].

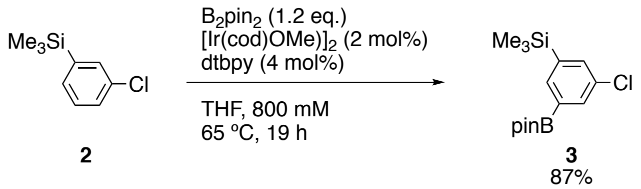

*3-Chloro-5-(trimethylsilyl)phenylboronic acid pinacol ester (**3**)*: A mixture of **2** (18.5 g, 100 mmol), $[Ir(cod)OMe]_2$ (1.32 g, 1.99 mmol), 4,4'-di-*t*-butyl-2,2'-bipyridine (1.08 g, 4.01 mmol) and bis(pinacolato)diboron (30.5 g, 120 mmol) in THF (125 mL) was stirred at 65 °C for 19 h. The solvent was removed in vacuo, and the residue was purified by silica gel chromatography (eluent: hexane). The title compound was obtained as a white powder in 87% yield (27.4 g, 88.2 mmol). ^{1}H NMR (600 MHz, $CDCl_3$): δ 7.78 (dd, J = 1.2, 0.6 Hz, 1H), 7.75 (dd, J = 2.4, 0.6 Hz, 1H), 7.53 (dd, J = 2.4, 1.2 Hz, 1H), 1.35 (s, 12H), 0.28 (s, 9H); ^{13}C NMR (150 MHz, $CDCl_3$): δ 142.6, 137.5, 136.0, 135.0, 134.1, 84.2, 25.0, –1.0 (A signal for the carbon nuclei bonded to the boron nuclei was not observed due to the quadrupolar relaxation induced by the boron nuclei.); HRMS (APCI) (*m*/*z*): $[M+H]^+$ calcd. for $C_{15}H_{25}BClO_2Si$ 310.1436, found 310.1427.

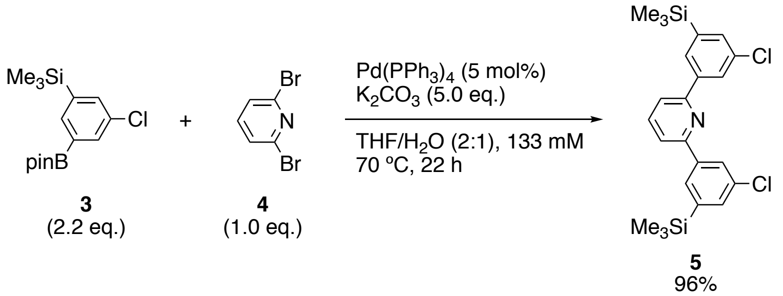

*3,3''-dichloro-5,5''-di(trimethylsilyl)-1,2':6',1''-terphenyl (**5**):* To a mixture of **3** (5.47 g, 17.6 mmol), 2,6-dibromopyridine (**4**) (1.90 g, 8.00 mmol) and $Pd(PPh_3)_4$ (464 mg, 0.402 mmol) in THF (40 mL) was added a solution of K_2CO_3 (3.46 g, 25.0 mmol) in water (20 mL). The mixture was stirred at 70 ºC for 22 h. After cooling down to an ambient temperature, the mixture was extracted with ethyl acetate (150 mL × 3), and the combined organic layer was dried over Na_2SO_4 and concentrated in vacuo. The crude material was purified by silica gel chromatography (eluent: hexane/ethyl acetate: 20:1). The title compound was obtained as a white powder in 96% yield (3.42 g, 7.69 mmol). ^{1}H NMR (600 MHz, $CDCl_3$): δ 8.16 (dd, J = 1.8, 1.2 Hz, 2H), 8.06 (dd, J = 1.8, 1.8 Hz, 2H), 7.85 (t, J = 7.8 Hz, 1H), 7.70 (d, J = 7.8 Hz, 2H), 7.53 (dd, J = 1.8, 1.2 Hz, 2H) 0.35 (s, 18H); ^{13}C NMR (150 MHz, $CDCl_3$): δ 156.0, 143.7, 140.5, 137.9, 134.8, 133.8, 129.9, 127.6, 119.4, –1.0; HRMS (APCI) (*m*/*z*): $[M+H]^+$ calcd. for $C_{23}H_{28}Cl_2NSi_2$ 444.1132, found 444.1136.

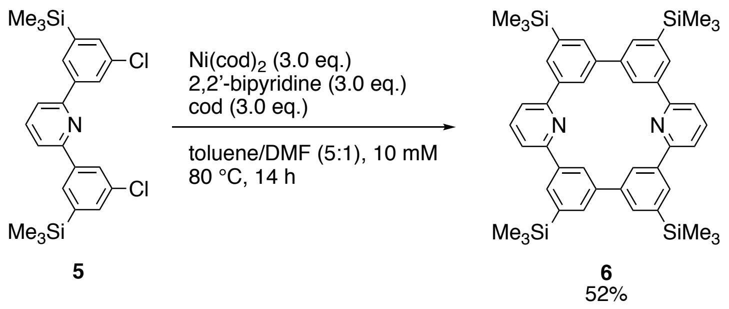

N-doped [6]cyclo-meta-phenylene derivative **6**: A mixture of 2,2'-bipyridine (2.35 g, 15.0 mmol), 1,5-cyclooctadiene (1.63 g, 15.1 mmol) and Ni(cod)$_2$ (4.16 g, 15.1 mmol) in toluene (83 mL) and DMF (83 mL) was stirred at 80 °C for 1 h. To the mixture was added a solution of terphenyl **5** (2.22 g, 5.00 mmol) in toluene (333 mL) dropwise, and the mixture was stirred at 80 °C for 14 h. The mixture was cooled down to ambient temperature and was stirred overnight after the addition of aqueous solution of HCl (1 M, *ca.* 320 mL). The aqueous layer was extracted with chloroform (200 mL × 3), and the combined organic layer was washed with sat. NaHCO$_3$ aq. (200 mL), dried over Na$_2$SO$_4$, and concentrated in vacuo. The crude solid was washed with hexane. The title compound was obtained as a white solid in 52% yield (963 mg, 1.29 mmol). ^{1}H NMR (600 MHz, CDCl$_3$): δ 9.18 (s, 4H), 8.02 (s, 4H), 7.90 (s, 4H), 7.89–7.84 (m, 6H), 0.40 (s, 36H); ^{13}C NMR (150 MHz, CDCl$_3$): δ 156.9, 141.2, 141.1, 139.2, 137.6, 132.6, 130.2, 127.6, 118.7, –0.8; HRMS (MALDI-TOF) (*m*/*z*): [M+H]$^+$ calcd. for $C_{46}H_{55}N_2Si_4$ 747.3437, found 747.3446.

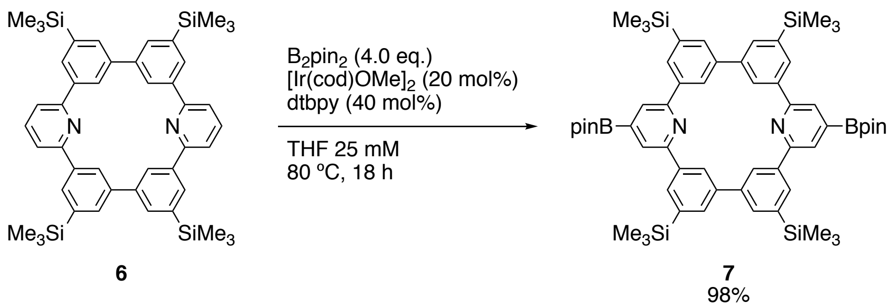

Borylated N-doped [6]CMP derivative **7**: A mixture of [6]CMP **6** (935 mg, 1.25 mmol), [Ir(cod)OMe]$_2$ (165 mg, 0.25 mmol), 4,4'-di-*t*-butyl-2,2'-bipyridine (134 mg, 0.50 mmol) and bis(pinacolato)diboron (1.26 g, 4.98 mmol) in THF (50 mL) was stirred at 80 °C for 18 h. The solvent was removed in vacuo, and the residue was washed with methanol (40 mL). After the material was dissolved in CHCl$_3$ (5 mL), methanol (60 mL) was added to lead the precipitate, which was collected by filtration. The title compound was obtained in 98% yield (1.22 g, 1.22 mmol). ^{1}H NMR (600 MHz, CDCl$_3$): δ 9.12 (dd, J = 1.8, 1.8 Hz), 8.14 (s, 4H), 8.07 (dd, J = 1.8, 0.9 Hz), 7.89 (dd, J = 1.8, 0.9 Hz), 1.46 (s, 24H), 0.41 (s, 36H); ^{13}C NMR (150 MHz, CDCl$_3$): δ 156.5, 141.1, 141.0, 139.4, 132.7, 130.3, 127.7, 123.7, 84.8, 25.1, –0.7 (A signal for the carbon nuclei bonded to the boron nuclei was not

observed due to the quadrupolar relaxation induced by the boron nuclei.); HRMS (APCI) (*m*/*z*): $[M+H]^+$ calcd. for $C_{58}H_{77}B_2N_2O_4Si_4$ 997.5214, found 997.5208.

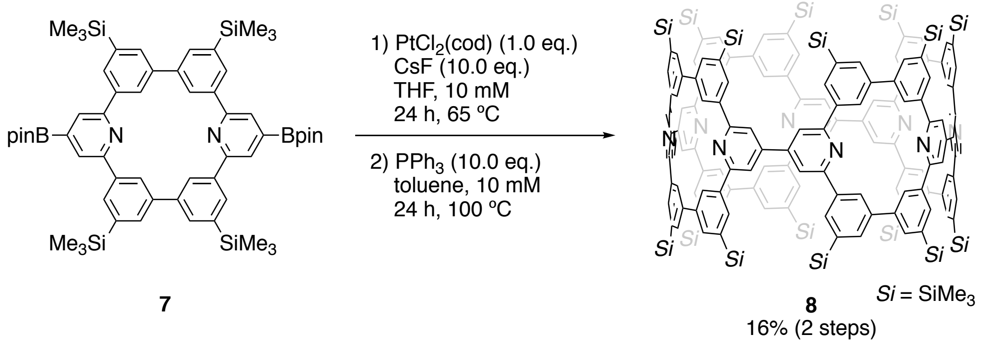

Macrocyclic N-doped [6]CMP derivative ***8***: A mixture of borylated [6]CMP **7** (100 mg, 0.100 mmol), $PtCl_2$(cod) (37.6 mg, 0.100 mmol) and CsF (151 mg, 0.994 mmol) in THF (10 mL) was stirred at 65 ºC for 24 h. The solvent was removed in vacuo, and the residue was diluted with toluene (10 mL). After addition of PPh_3 (264 mg, 1.01 mmol), the mixture was stirred at 100 ºC for 24 h. The mixture was poured to methanol (100 mL) to afford a crude solid material. The crude material was filtrated, taken up in $CHCl_3$ (10 mL) and subjected to GPC to afford the title compound in 16% yield (11.9 mg, 39.9 μmol). ^{1}H NMR (600 MHz, $CDCl_3$): δ 8.88 (s, 16H), 8.09 (s, 16H), 7.94 (s, 16H), 7.93 (s, 16H), 0.41 (s, 144H); ^{13}C NMR (150 MHz, $CDCl_3$): δ 160.0, 146.7, 141.6, 140.4, 138.2, 130.6, 130.3, 128.0, 117.5, –0.8; HRMS (MALDI-TOF) (*m*/*z*): $[M+H]^+$ calcd. for $C_{184}H_{209}N_8Si_{16}$ 2978.2903, found 2978.2957.

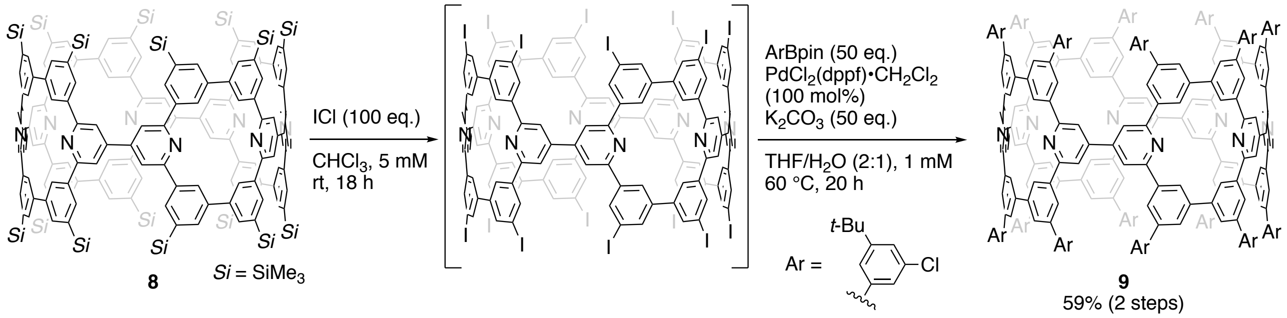

Macrocyclic N-doped [6]CMP derivative ***9***: To a solution of **8** (44.7 mg, 15.0 μmol) in $CHCl_3$ (1.5 mL) was added a solution of ICl (245 mg, 1.51 mmol) in $CHCl_3$ (1.5 mL). The mixture was stirred at ambient temperature for 18 h. To the mixture was added saturated $Na_2S_2O_3$ aq. (*ca*. 10 mL), and the precipitates were collected by filtration. The solid was washed with $CHCl_3$ and methanol to give iodinated product as a white powder. The crude iodinated compound was then mixed with 3-chloro-5-*t*-butyl-phenylboronic acid pinacol ester (233 mg, 751 μmol) and $PdCl_2$(dppf)·DCM (13.1 mg, 16.0 μmol). After the addition of THF (10.0 mL) and a solution of K_2CO_3 (105 mg, 0.757 μmol) in water (5 mL), the mixture was stirred at 60 °C for 20 h. The reaction mixture was cooled down to

an ambient temperature, and the organic materials were extracted by $CHCl_3$ (50 mL × 3), dried over Na_2SO_4 and concentrated in vacuo. The crude material was purified by GPC to give the title compound as a yellow powder in 59% yield (40.0 mg, 8.9 μmol). ^{1}H NMR (600 MHz, $CDCl_3$): ^{1}H NMR (600 MHz, $CDCl_3$): *δ* 8.99 (s, 16H), 8.13 (s, 16H), 8.02 (s, 16H), 7.97 (s, 16H), 7.55 (dd, *J* = 1.8, 1.8 Hz, 16H), 7.51 (dd, *J* = 1.8, 1.8 Hz, 16H), 7.41 (dd, *J* = 1.8, 1.8 Hz, 16H), 1.36 (s, 144H); ^{13}C NMR (150 MHz, $CDCl_3$): *δ* 159.7, 154.1, 147.5, 142.7, 142.3, 141.5, 138.9, 134.8, 126.6, 125.3, 125.2, 125.0, 125.0, 123.0, 118.4, 35.2, 31.4; MS (MALDI-TOF) (*m*/*z*): $[M+H]^+$ calcd. for $C_{296}H_{257}Cl_{16}N_8$ 4492.5, found 4492.8.

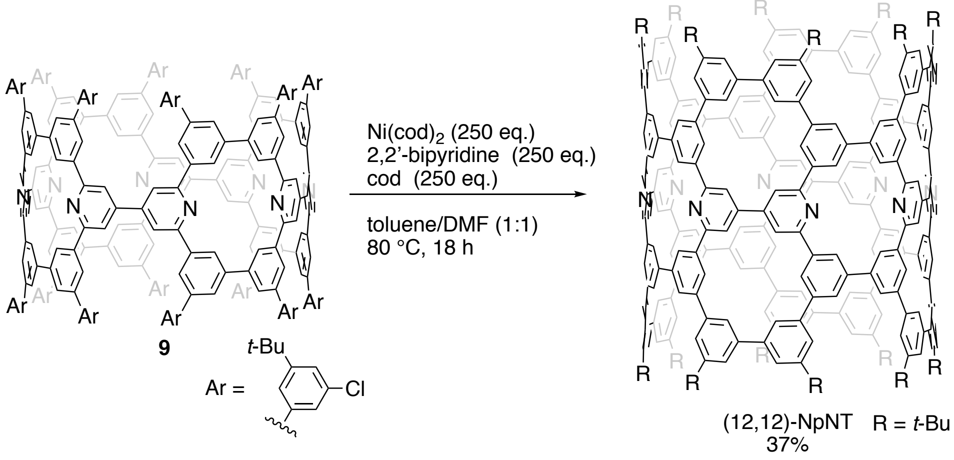

(12,12)-NpNT: A mixture of 2,2'-bipyridine (391 mg, 2.50 mmol), 1,5-cyclooctadiene (273 mg, 2.53 mmol) and $Ni(cod)_2$ (695 mg, 2.53 mmol) in DMF (5 mL) was stirred at 80 ºC for 40 min. To the mixture was added a solution of compound **9** (45 mg, 10 μmol) in toluene (5 mL), and the reaction mixture was stirred at 80 ºC for 18 h. The mixture was then cooled to ambient temperature and was further stirred for additional 6 h after addition of saturated NH_4Cl aq. (ca. 15mL). The aqueous layer was extracted with toluene (70 mL × 3), and the combined organic layer was washed with brine, dried over anhydrous Na_2SO_4 and concentrated in vacuo. The crude material was purified by GPC to afford the title compound as a white solid in 37% yield (14 mg, 3.7 μmol). ^{1}H NMR (600 MHz, $CDCl_3$): *δ* 9.38 (s, 16H), 8.49 (s, 16H), 8.45 (s, 16H), 8.33 (s, 16H), 8.16 (s, 16H), 8.01 (s, 16H), 7.89 (s, 16H), 1.55 (s, 144H); ^{13}C NMR (150 MHz, $CDCl_3$): *δ* 159.3, 152.8, 143.8, 142.7, 142.1, 140.4, 138.4, 137.5, 126.2, 124.2, 124.0, 123.8, 123.2, 121.3, 117.5, 35.4, 31.7; HRMS (MALDI-TOF) (*m*/*z*): $[M+H]^+$ calcd. for $C_{296}H_{257}N_8$ 3923.0351, found 3923.0420.

Locations of Defects

The defects in the NpNT molecule can be categorized into four types: replaced atom, depleted atom, replaced bond and depleted bond (Supplementary Fig. 1). The locations of nitrogen atoms and atom vacancy defects are designated as coordinates in the oblique coordinate system of nanotubes[4], and the details are described as follows. The present coordinate system is developed by adding a few new factors to the previous vector nomenclatures of finite carbon nanotube molecules. The previous geometric descriptors of finite nanotube molecules, such as bond-filling index (F_b), atom-filling index (F_a) and length index (t_f), are also integrated in the present system[5].

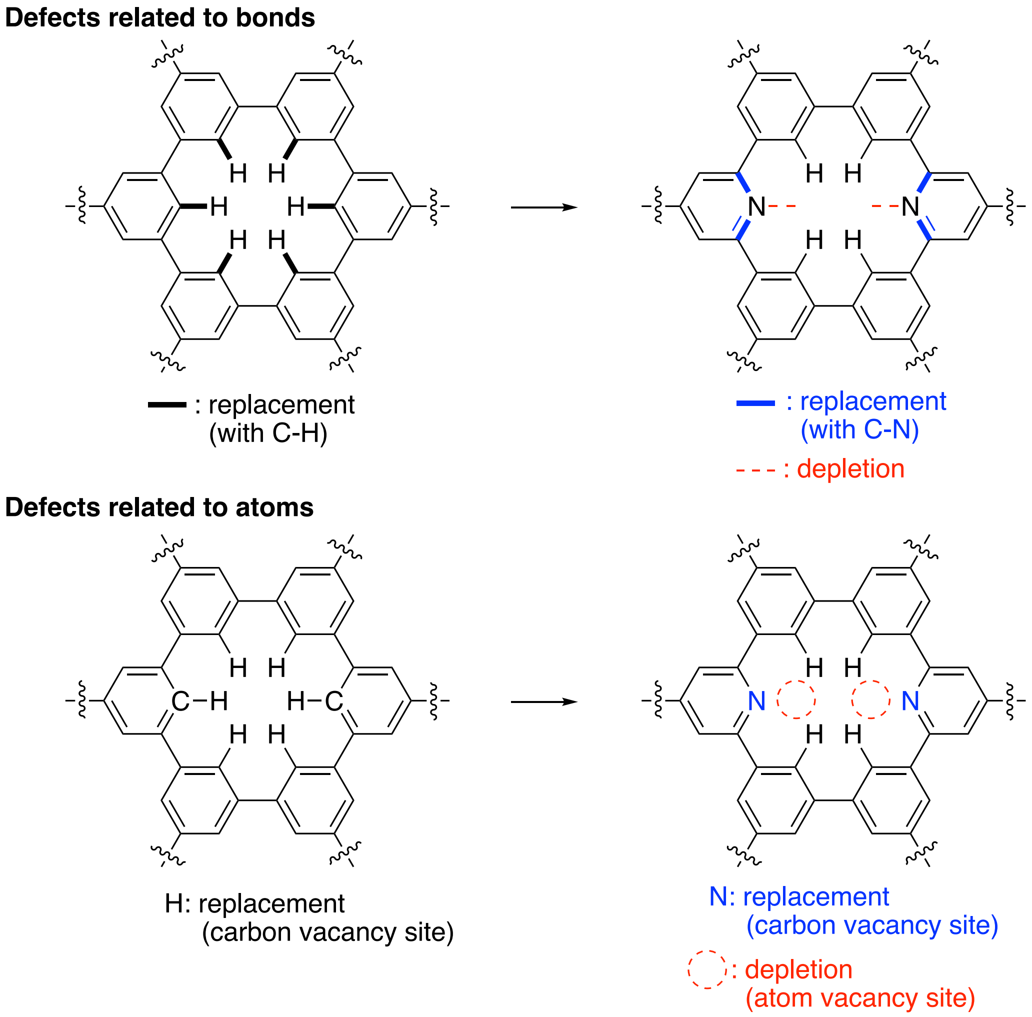

Supplementary Fig. 1 | Types and names of defects.

Most of quantitative measures of defects have been established in the previous geometric indices such as t_f = 7.0, F_b = 53.49% and F_a = 64.44% for (12,12)-NpNT[5,6], and the descriptors of defect locations are defined in this study. The oblique coordinate system of nanotubes has already been developed[4], and defect locations can be designated by setting the point of origin of the coordinate in the finite nanotube system. The origin should be located at the edge of the nanotube

molecule[7], and atoms located at the edge are the candidate of the origin. Edge atoms at both ends of the nanotube should commonly be compared. When there exist multiple edge atoms, we must compare the candidates with priority rules, and the IUPAC priority rules are adopted. The higher priority is thus given to an atom with the higher atomic number, and the highest-priority edge atom should be assigned as the origin. When we have multiple edge atoms with the identical highest atomic number, the priority of non-edge atoms should then be considered, and an edge atom that is located closest to the highest-priority non-edge atom should be assigned as the origin[8]. When we set the origin of the coordinate on the (12,12)-NpNT molecule by following this selection rule, we can fully designate defects with the coordinates shown in Supplementary Fig. 2[9]. A web applet for the geometric measures [10] has been developed and provided at https://physorg.chem.s.u-tokyo.ac.jp/applet/defect/ .

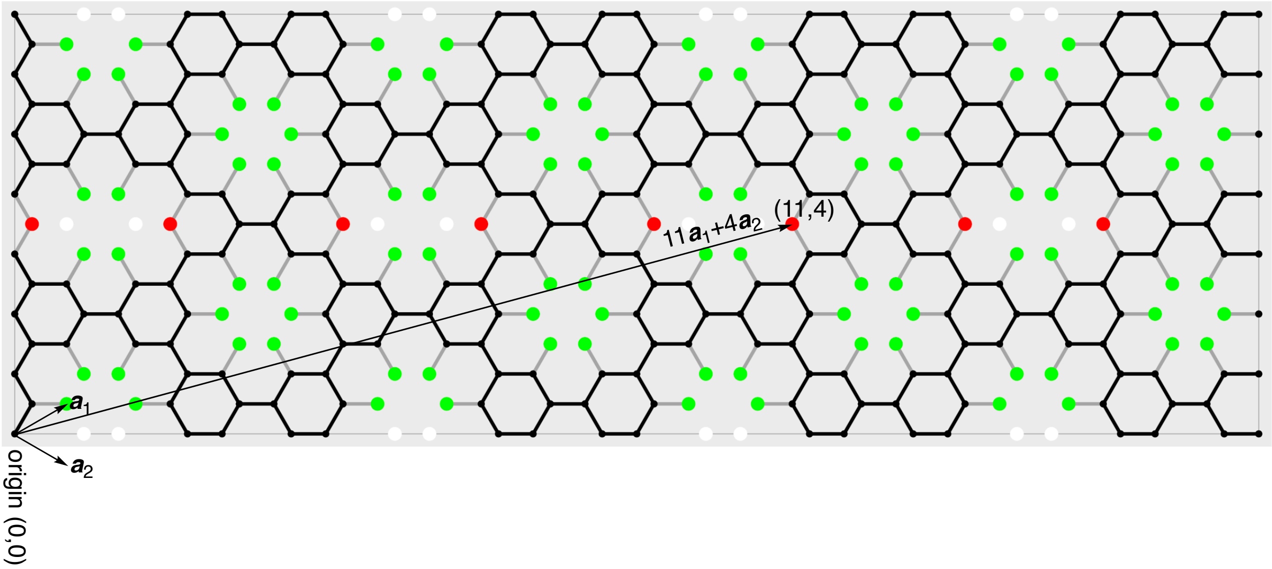

●: replaced atom (N) ●: replaced atom (H) ○: depleted atom (atom vacancy site) ▬: replaced bond

$\boldsymbol{a}_1$ and $\boldsymbol{a}_2$: unit vector

length index t_f = 7.0, bond-filling index F_b = 53.49%, atom-filling index F_a = 64.44%

coordinate of N: (5, -2), (8, 1), (11/3, -10/3), (11, 4), (14, 7), (20/3, -1/3), (29/3, 8/3), (38/3, 17/3)

coordinate of H: (1, 0), (2, 0), (4, 0), (4, 1), (4, 3), (4, -2), (5/3, 2/3), (5/3, -1/3), (5, 0), (5, 3), (5, -3), (7, 1), (7, 3), (7, 4), (7, 6), (7, -2), (7, -3), (7, -5), (7, -6), (8, 0), (8, 3), (8, 6), (8, -3), (10, 0), (10, 1), (10, 4), (10, 6), (10, 7), (10, 9), (10, -2), (10, -3), (11/3, 2/3), (11/3, -7/3), (11, 0), (11, 3), (11, 6), (11, 9), (13, 0), (13, 1), (13, 3), (13, 4), (13, 7), (13, 9), (13, 10), (14/3, 2/3), (14/3, 8/3), (14/3, 11/3), (14/3, -1/3), (14/3, -10/3), (14, 3), (14, 6), (14, 9), (16, 3), (16, 4), (16, 6), (16, 7), (17, 6), (20/3, 2/3), (20/3, 11/3), (20/3, -7/3), (20/3, -16/3), (23/3, 8/3), (23/3, 11/3), (23/3, 17/3), (23/3, 20/3), (23/3, -1/3), (23/3, -7/3), (23/3, -10/3), (23/3, -16/3), (29/3, 2/3), (29/3, 11/3), (29/3, 20/3), (29/3, -7/3), (32/3, 2/3), (32/3, 8/3), (32/3, 17/3), (32/3, 20/3), (32/3, 26/3), (32/3, 29/3), (32/3, -1/3), (32/3, -7/3), (38/3, 2/3), (38/3, 11/3), (38/3, 20/3), (38/3, 29/3), (41/3, 2/3), (41/3, 8/3), (41/3, 11/3), (41/3, 17/3), (41/3, 26/3), (41/3, 29/3), (47/3, 11/3), (47/3, 20/3), (50/3, 11/3), (50/3, 17/3), (50/3, 20/3)

coordinagte of depleted atoms: (1, 1), (2/3, 2/3), (4, 4), (4, -3), (7, 0), (7, 7), (8, -6), (10, 3), (10, 10), (11/3, 11/3), (11, -3), (13, 6), (14/3, -7/3), (14, 0), (17, 3), (20/3, 20/3), (23/3, 2/3), (23/3, -19/3), (29/3, 29/3), (32/3, 11/3), (32/3, -10/3), (41/3, 20/3), (41/3, -1/3), (50/3, 8/3)

Supplementary Fig. 2 | Representative geometric measures of the (12,12)-NpNT molecule.

Crystallography

Initial IAM refinements with SHELXL (stage 0)

The initial structure was solved by a direct method by using the SHELXT software program[11]. A full-matrix least-squares on F^2 using the SHELXL (SHELX-2018/3)[12] was performed for a conventional IAM refinement *via* Yadokari-XG 2009 software program as an interface (Supplementary Fig. 3)[13]. At this stage, severe structural disorders were mainly found and located at *t*-butyl groups of the molecule. The solvent molecules were not modelled, and the structures were refined by using the PLATON/SQUEEZE protocol (version 220719)[14,15]. The non-hydrogen atoms were anisotropically refined with hydrogen atoms attached as riding models at calculated positions, and the disordered *t*-butyl structures were refined under restraints of SIMU, DFIX and DANG. The initial molecular structure of the (12,12)-NpNT molecule with *t*-butyl disorders was finalized with moderate yet acceptable *R* factors: $R_1(F^2) = 0.1267$ and $wR_2(F^2) = 0.3624$ $[I > 2\sigma(I)]$ (stage 0).

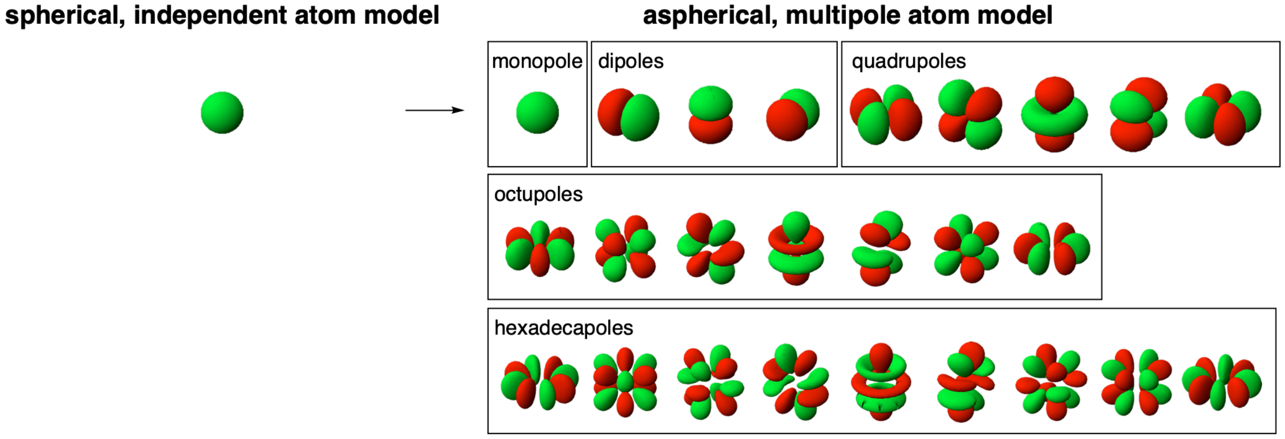

Supplementary Fig. 3 | Spherical independent atom models and aspherical atom models with multipoles.

Initial IAM and TAAM refinements with XD2016 (stage 1 and 2)

To refine the molecular structure with a higher accuracy, we decided to further refine the structure with multipole aspherical atom models (Supplementary Fig. 3), which unexpectedly located anomalous disordered structures. The moderate level of the present diffraction data was not suitable for empirical multipole aspherical models, and TAAM protocols were adopted [16]. The IAM/SHELXL-refined structure after the PLATON/SQUEEZE process of the solvent-density removal (version 270712) was transferred, along with the processed HKL data, to the XD2016 program for the IAM refinement. The structure was refined with IAM on XD2016 by a full-matrix least-squares on *F* with XDLSM (stage 1, Supplementary Fig. 4)[17]. All the parameters of the non-disordered structure were refined with no restraint while those of the disordered structures were

constrained to assure the convergence of the refinement. The resultant R factors were $R(F) = 0.1010$ and $Rw(F) = 0.1533$.

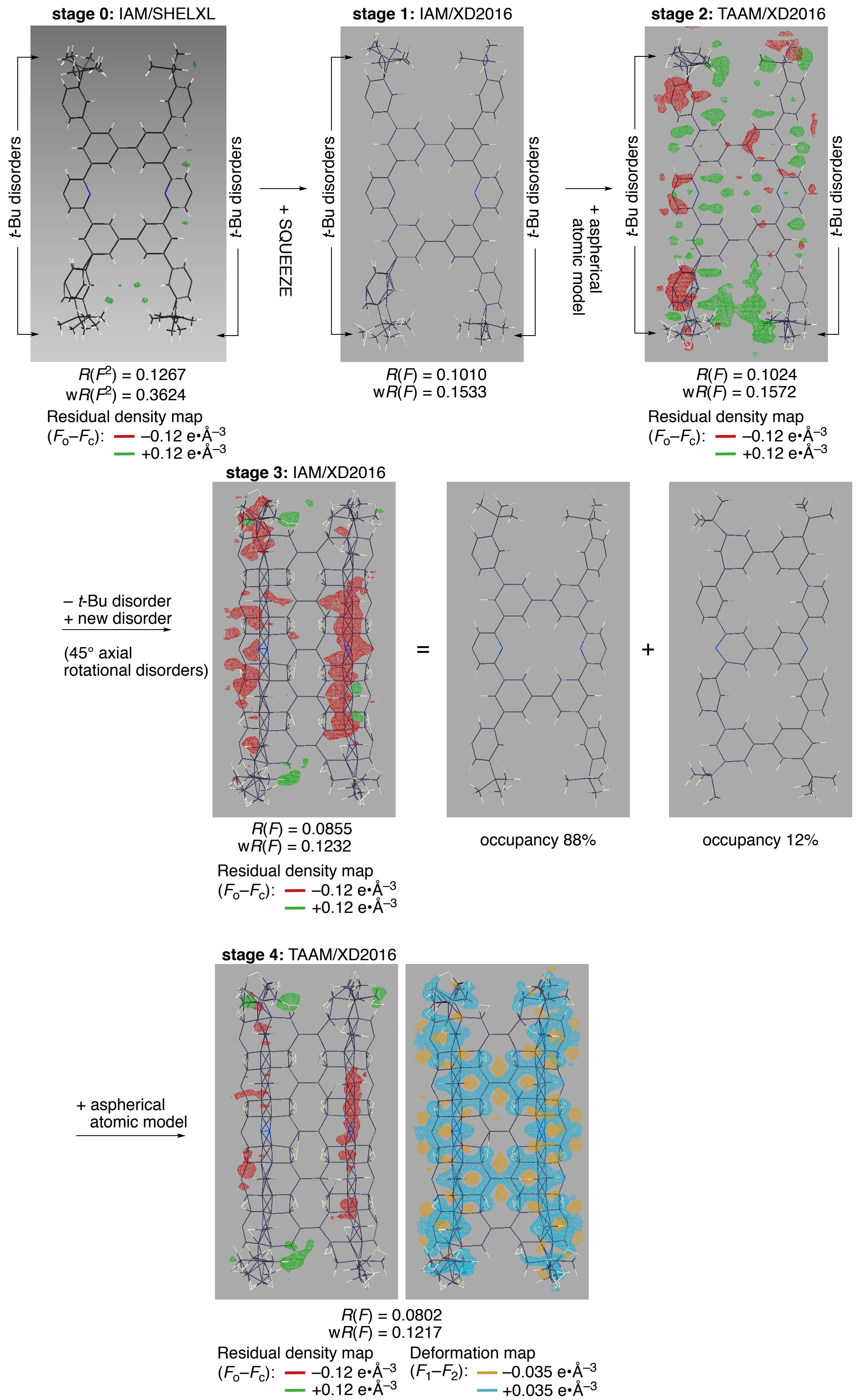

Supplementary Fig. 4 | Model structures in the refinement.

The TAAM refinement incorporating aspherical atom models was then carried out using the parameters of the University at Buffalo Pseudoatom Databank (UBDB)[18,19]. The multipole parameters of P_v, P_{lmp}, κ and κ' were transferred from the UBDB and used without refinements. All the parameters of the non-disordered structure were refined with no restraint by a full-matrix least-squares on F with XDLSM, while those of the disordered structures were constrained to assure the convergence of the refinement. The TAAM refinements resulted in inferior R factors [$R(F)$ = 0.1024 and $Rw(F)$ = 0.1572] (stage 2, Supplementary Fig. 4), which urged us to closely examine the residual electron density in details. The resultant residual electron density ($F_o - F_c$) after the TAAM refinements is shown in Supplementary Fig. 4. Interestingly, the unassigned, positive residual densities were found at the atom vacancy defects, and we realized that the refined structure might not be correct, particularly, at the disorder assignments.

Final IAM and TAAM refinements with SHELXL and XD2016 (stage 3 and 4)

Examining the unassigned electron densities, we noticed a possibility of an unusual disordered structure. The residual density around the atom vacancy defects may thus imply the presence of 6 atoms at the biaryl linkage, which could be originated from an axially rotated structure of the cylindrical molecule. Locating this new unique disordered structure as a minor disordered structure, we restarted the structural refinement by a full-matrix least-squares on F^2 first with IAM/SHELXL *via* the PLATON/SQUEEZE solvent treatments. With SIMU, DFIX and DANG restraints on the rotated minor structure (occupancy = 12%), the R factors were improved: $R_1(F^2)$ = 0.1049 and $wR_2(F^2)$ = 0.3261 [$I > 2\sigma(I)$]. The structure and the PLATON/SQUEEZE-processed HKL data were then transferred to XD2016, and the IAM refinement by a full-matrix least-squares on F with XDLSM were performed. All the parameters of the major 88%-structure were refined, while those of the minor 12%-structure were fixed to assure the convergence, and the refinements resulted in an improved R factors: $R(F)$ = 0.0855 and $Rw(F)$ = 0.1232 (stage 3, Supplementary Fig. 4).

The aspherical multipole models were finally applied to the new disordered structure by using TAAM protocols with UBDB parameters. For the accurate refinement, hydrogen atoms were relocated by extending X–H distances to their standard neutron diffraction values[19]. The major 88%-structure was fully refined, and the 12%-minor structure was fixed with constraints to assure the convergence. This final refinement by a full-matrix least-squares on F resulted in the improved R factors: $R(F)$ = 0.0802 and $Rw(F)$ = 0.1217 (stage 4; Supplementary Fig. 4 and Supplementary Table 1). Comparisons of the residual electron density ($F_o - F_c$) between stage-3 and stage-4 refinements showed that the incorporations of the aspherical models reduced the unassigned densities. The electron densities were mainly located on the 88%-major structure, and the deformation map ($F_1 - F_2$)

revealed the effects of chemical bonds over the major structure (Supplementary Fig. 4). The diameters of the cylinders were nearly uniform and were also identical between two disordered structures (Supplementary Fig. 5). When compared with (12,12)-pNT,[20] the shape and size of the cylinder were not affected by the nitrogen dopants (Supplementary Fig. 6)

Visualization and analyses

Molecular structures were visualized and analyzed by Mercury CSD 4.1.0[21] and UCSF Chimera (ver. 1.13.1)[22]. The electron densities after TAAM/XD2016 refinements were visualized by MoleCoolQt[23]. The contour map of the deformation density was obtained by the XDGRAPH module in the XD2016 package[17], and the ESP map was generated by MolIso[24].

Supplementary Table 1 | Crystal data and structure refinement for (12,12)-NpNT *via* TAAM/XD2016.

Data deposition	CCDC 1966650	
Empirical formula	$C_{296}H_{256}N_8$	
Formula weight	3925.07	
Temperature	100(2) K	
Wavelength	0.83000 Å	
Crystal system	Monoclinic	
Space group	*C*2/*m*	
Unit cell dimensions	a = 37.140(7) Å	α = 90°
	b = 28.020(6) Å	β = 98.36(3)°
	c = 24.020(5) Å	γ = 90°
Volume	24731(9) Å^3	
Z	2	
Density (calculated)	0.527 g/cm^3	
Absorption coefficient	0.041 mm^{-1}	
F(000)	4176	
Crystal size	0.04 × 0.03 × 0.02 mm^3	
Theta range for data collection	1.067 to 29.986°	
Limiting indices	$-44<=h<=44$, $-33<=k<=33$, $-28<=l<=28$	
Independent reflections	22969 [R(int) = 0.0638]	
Completeness to theta = 29.868°	99.5 %	
Absorption correction	Semi-empirical from equivalents	
Max. and min. transmission	1.000 and 0.858	

Refinement method	Full-matrix least-squares on *F*
Data / restraints / parameters	22969 / 0 / 667
Goodness-of-fit (GOFw)	1.187
Final *R* indices	*R*(*F*) = 0.0802, *Rw*(*F*) = 0.1217
	$R(F^2)$ = 0.1356, $Rw(F^2)$ = 0.2451
Extinction coefficient	n/a

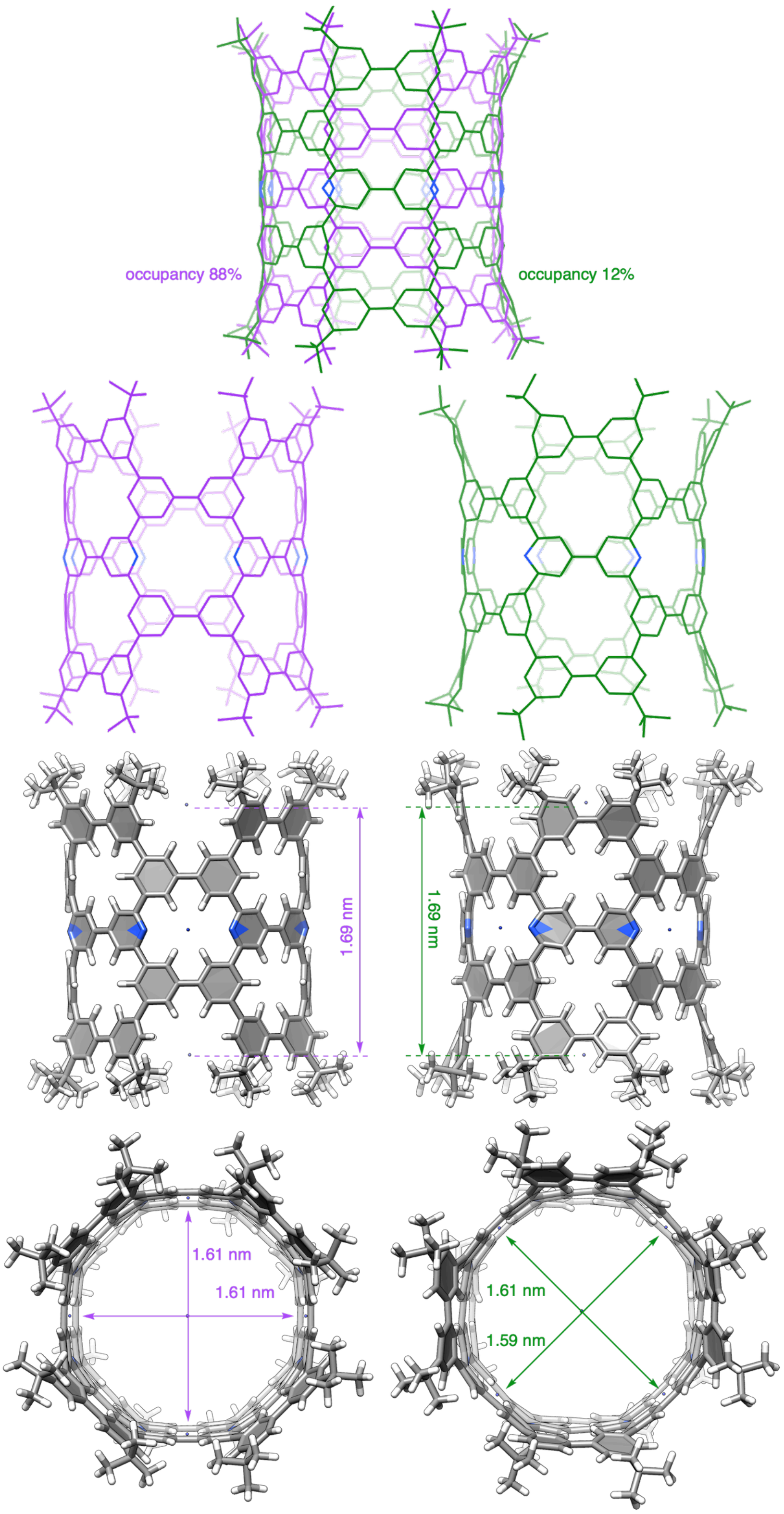

Supplementary Fig. 5 | Sizes of (12,12)-NpNT from crystal structures. For the measurement, we set two centroids of 16 sp^2-carbon atoms at the rim of the nanotube and four centroids of 4 hydrogen atoms at the circumferential [6]CMP openings. The length is measured as the distance between two centroids at the rim, and the diameter is measured as the distance between four centroids at the circumference. The methods of measurements were identical to those adopted for (12,12)-pNT[20].

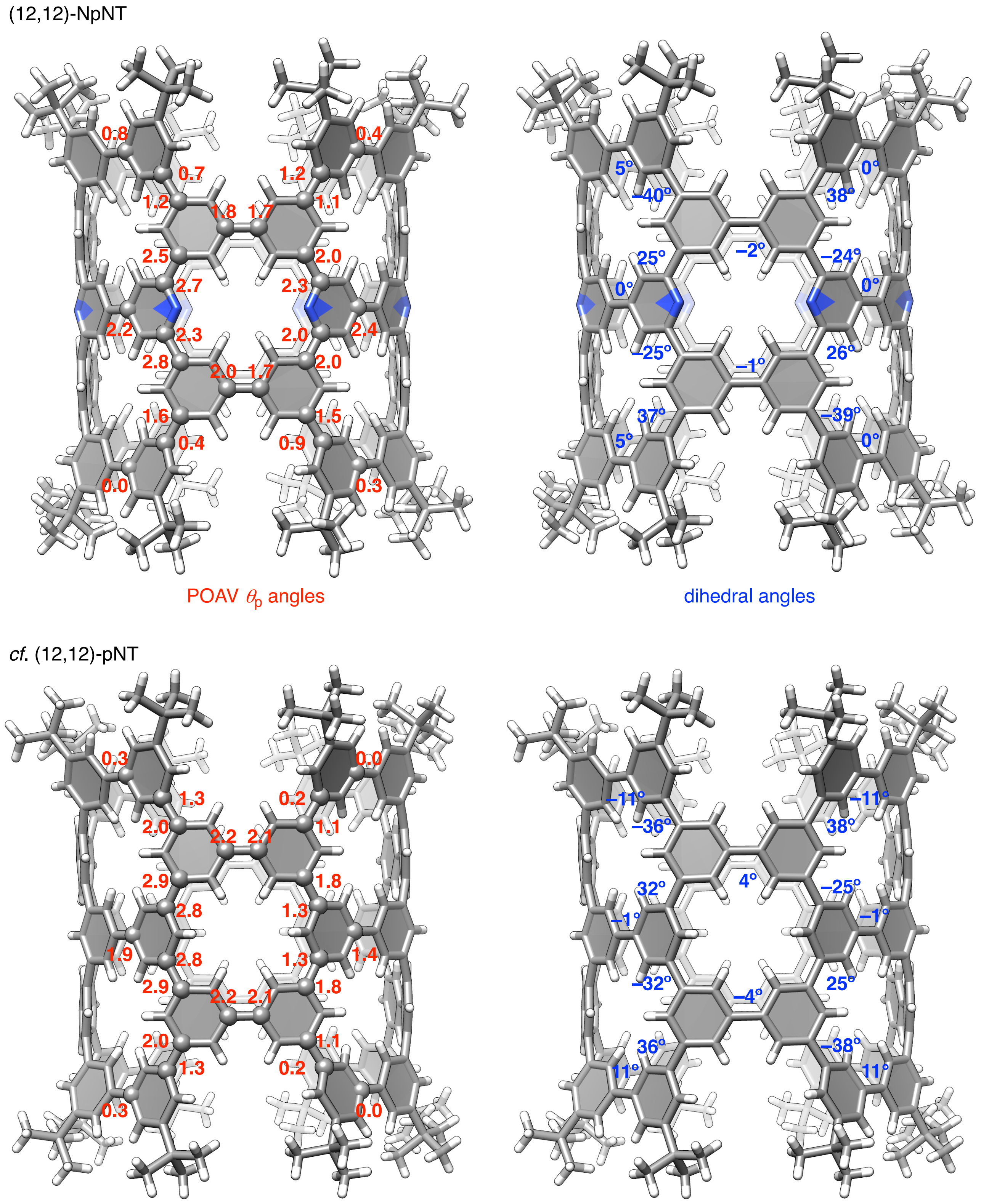

Supplementary Fig. 6 | POAV θ_p angles and dihedral angles of (12,12)-NpNT from crystal structures. Reference values of (12,12)-pNT are also shown. The methods of measurements were identical to those adopted for (12,12)-pNT[20].

Diffraction experiments of a single crystal of 4,4'-bipyridine

A single crystal of 4,4'-bipyridine suitable for X-ray crystallographic analysis was grown in hexane at 25 °C. A single crystal was mounted on a thin polymer tip with cryoprotectant oil and frozen at –173 °C via flash-cooling. The data collection was carried out on a Rigaku XtaLAB P200 diffractometer equipped with a PILATUS200K detector using multi-layer mirror monochromated Cu-Kα radiation with the resolution up to sin $\theta \bullet \lambda^{-1}$ = 0.60 Å^{-1}. CrystalClear[25] and CrysAlisPro[26] were used for the data collection and processing, respectively. The structure was first solved by the direct method with SHELXT program[11] and refined by full-matrix least-squares on F^2 using the SHELXL program suite[12] running on the Yadokari-XG 2009 software program[13]. In the refinements, the non-hydrogen atoms were analyzed anisotropically, hydrogen atoms of 4,4'-bipyridine were input at the calculated positions and refined with a riding model. Hydrogen atoms of water molecules were located by referring to the residual electron density maps and were restrained by DFIX and DANG to afford $R_1(F^2)$ = 0.0275 and $wR_2(F^2)$ = 0.0778 [$I > 2\sigma(I)$]. The crystal structure was essentially identical to the previously reported structure refined by the IAM/SHELXL protocol[27]. The aspherical multipole models were then applied to the structure by using TAAM protocols[16] with UBDB parameters[18]. The refinement was performed by a full-matrix least-squares on F with the XDLSM module implemented on the XD2016 package[17]. In the refinement, hydrogen atoms were relocated by extending X–H distances to their standard neutron diffraction values. The refinement resulted in the improved R factors: $R(F)$ = 0.0198 and $Rw(F)$ = 0.0282 (Supplementary Table 2). Comparisons of the bond lengths with (12,12)-NpNT are shown in Supplementary Fig. 7, and a deformation density map and an electrostatic potential map are shown in Supplementary Fig. 8. The charge density analyses of 4,4'-bipyridine afforded crystallographic data (deformation density map and electrostatic potential map) similar to those of (12,12)-NpNT, which also supported the validity of the SQUEEZE/TAAM procedures devised for NpNT.

Supplementary Table 2 | Crystal data and structure refinement for 4,4'-bipyridine *via* TAAM/XD2016.

Data deposition	CCDC 1966651
Empirical formula	$C_{10}H_8N_2$, 2(H_2O)
Formula weight	192.22
Temperature	93 K
Wavelength	1.54184 Å
Crystal system	Monoclinic
Space group	$P2_1$
Unit cell dimensions	a = 9.12142(6) Å α = 90.0000°

	b = 7.41040(5) Å β = 100.9913(6)°
	c = 14.72097(10) Å γ = 90.0000°
Volume	976.787(12) $Å^3$
Z	4
Density (calculated)	1.307 g/cm^3
Absorption coefficient	0.763 mm^{-1}
F(000)	408.00
Crystal size	0.20 × 0.07 × 0.05 mm^3
Theta range for data collection	3.058 to 67.684°
Limiting indices	$-10<=h<=10$, $-8<=k<=8$, $-17<=l<=17$
Reflections collected	65832
Independent reflections	3535 [R(int) = 0.0434]
Completeness to theta = 67.684°	99.9 %
Absorption correction	Semi-empirical from equivalents
Max. and min. transmission	0.963 and 0.698
Refinement method	Full-matrix least-squares on F
Data / restraints / parameters	3535 / 13 / 277
Goodness-of-fit (GOFw)	0.755
Final R indices	$R(F)$ = 0.0198, $Rw(F)$ = 0.0282
	$R(F^2)$ = 0.0543, $Rw(F^2)$ = 0.0558
Extinction coefficient	n/a

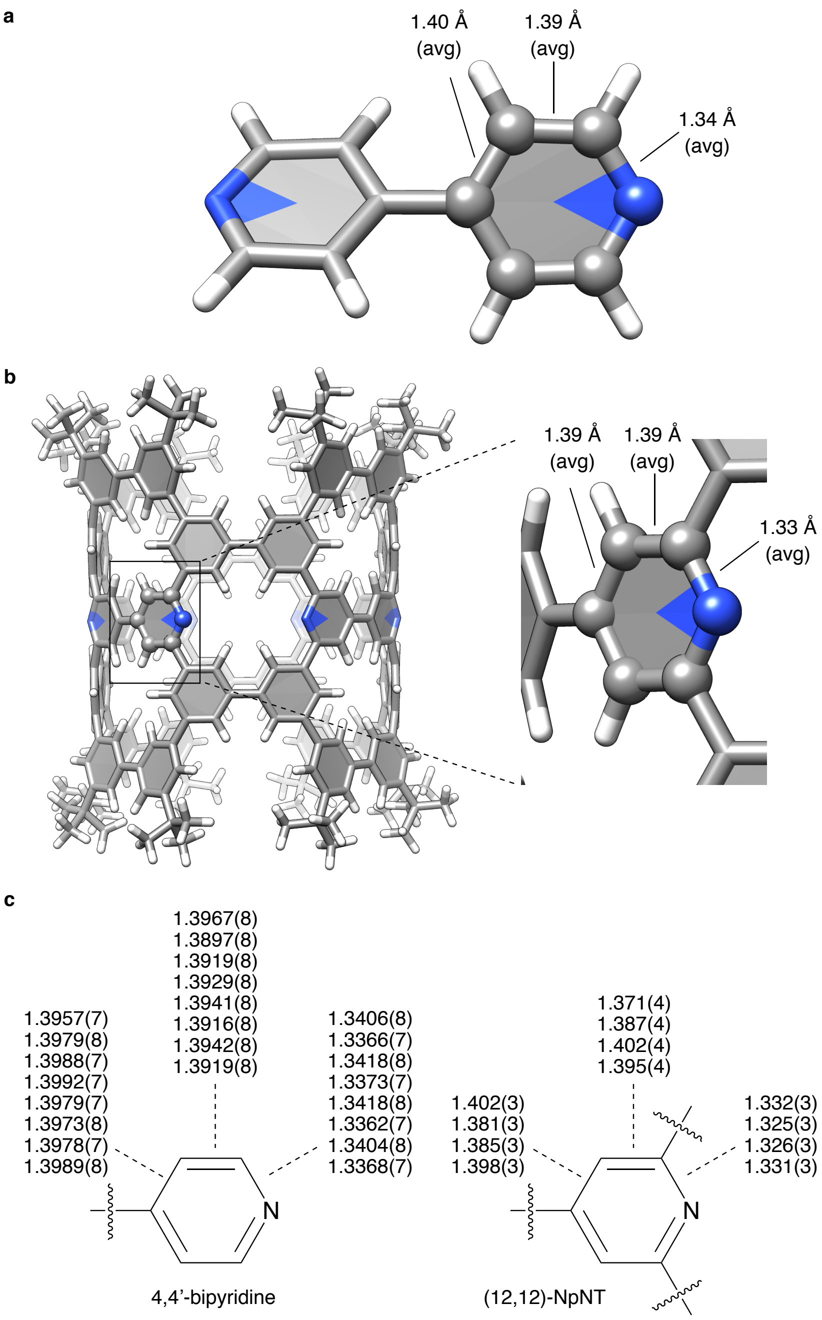

Supplementary Fig. 7 | Bond lengths. a, 4,4'-bipyridine. **b**, (12,12)-NpNT. **c,** Raw bond-length data.

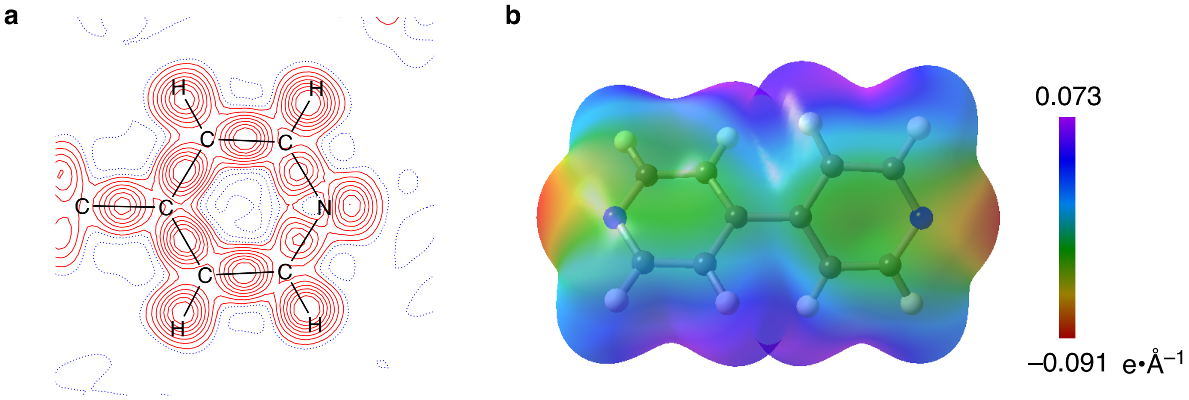

Supplementary Fig. 8 | Charge density analyses with 4,4'-bipyridine. a, Deformation map (Contour interval: 0.06 e•$Å^{-3}$, positive: red, negative: blue). **b,** Electrostatic potential map mapped on the 0.0067 e•$Å^{-3}$ isosurface of the electron density. For comparisons of corresponding data of NpNT, see Fig. 3.

DFT Calculations

As was the case with (12,12)-pNT, the geometry optimizations of (12,12)-NpNT and 4,4'-bipyridine were performed at the PBEPBE/STO-3G level of theory[28,29] by using Gaussian 16 program suite[30]. Coordinates are provided in Supplementary Data 1 and Supplementary Data 2.

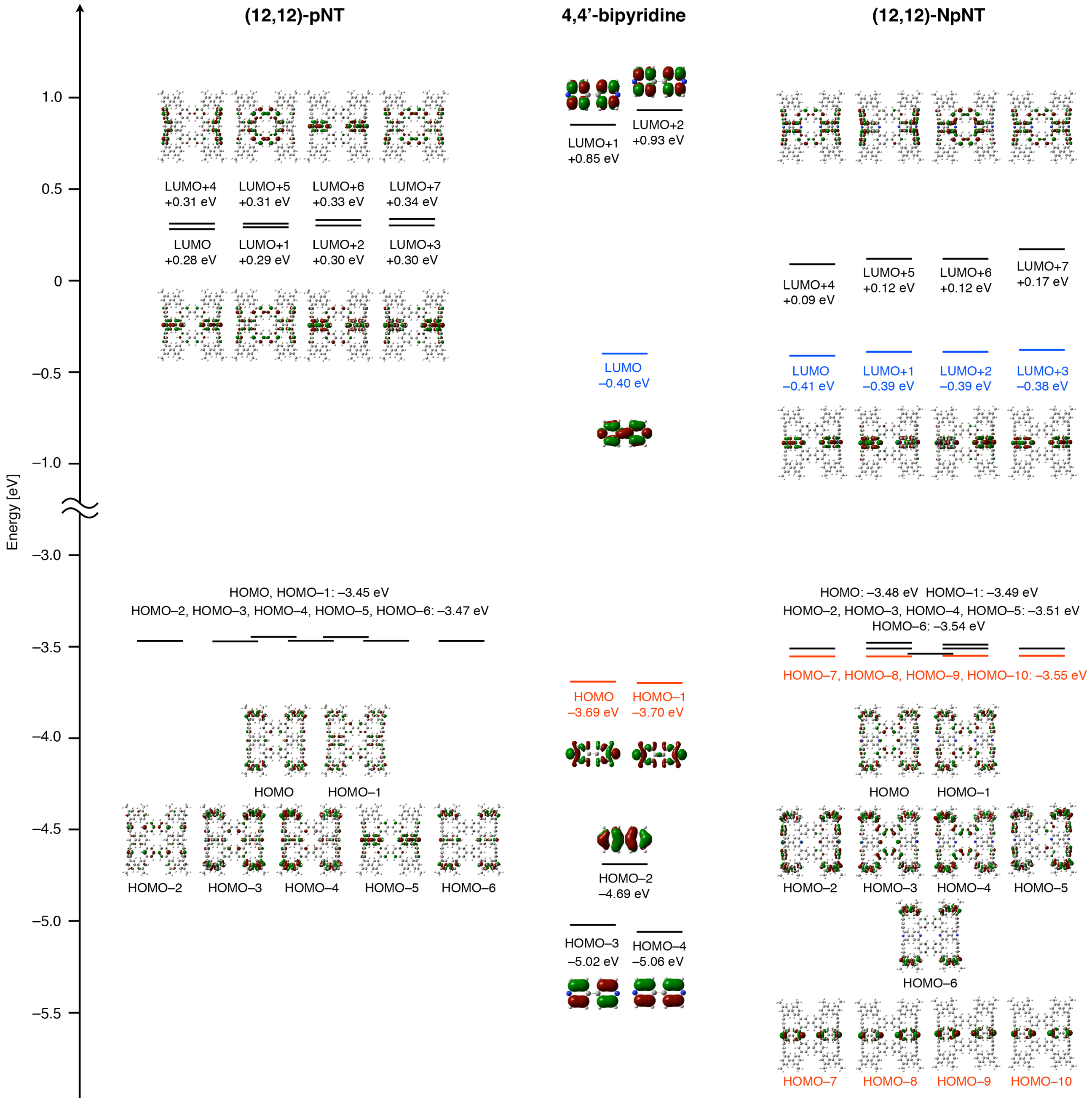

Supplementary Fig. 9 | Energy diagram of frontier Kohn-Sham orbitals of (12,12)-pNT, 4,4'-bipyridine and (12,12)-NpNT.

Chromatograms

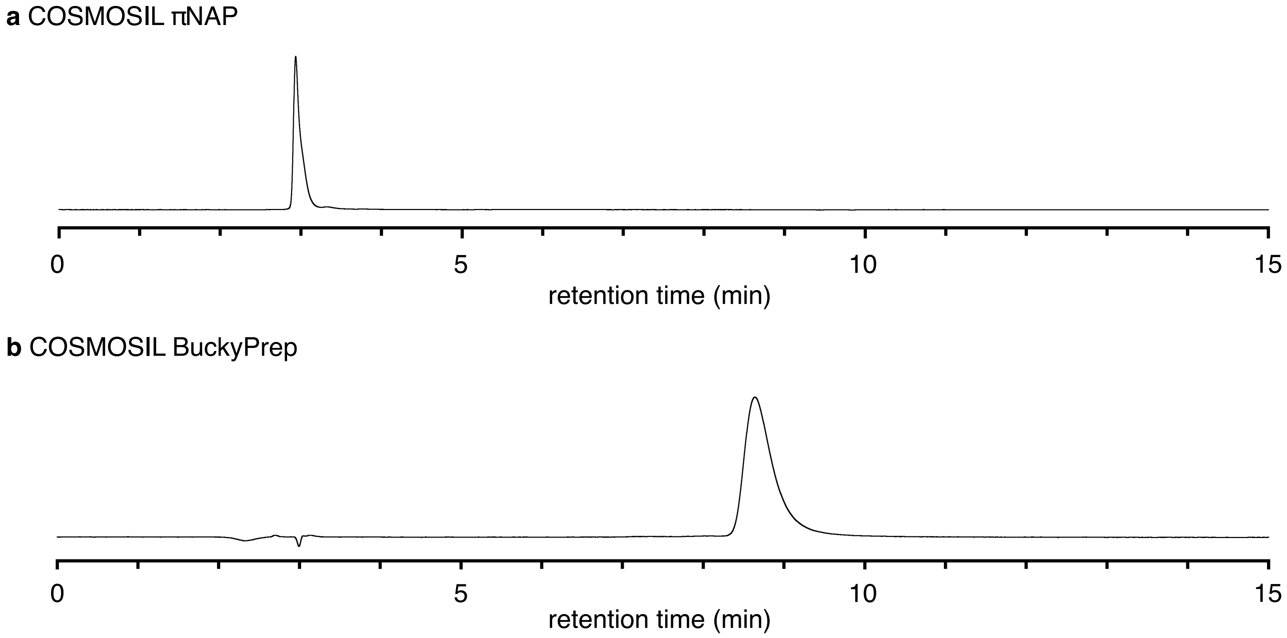

Supplementary Fig. 10 | Chromatograms of (12,12)-NpNT assuring the purity of (12,12)-NpNT. **a**, COSMOSIL πNAP. **b**, BuckyPrep. Chromatographic conditions: flow rate = 1.0 mL/min, eluent = $CHCl_3$/MeOH (7:3) and detection = 280 nm.

NMR spectra

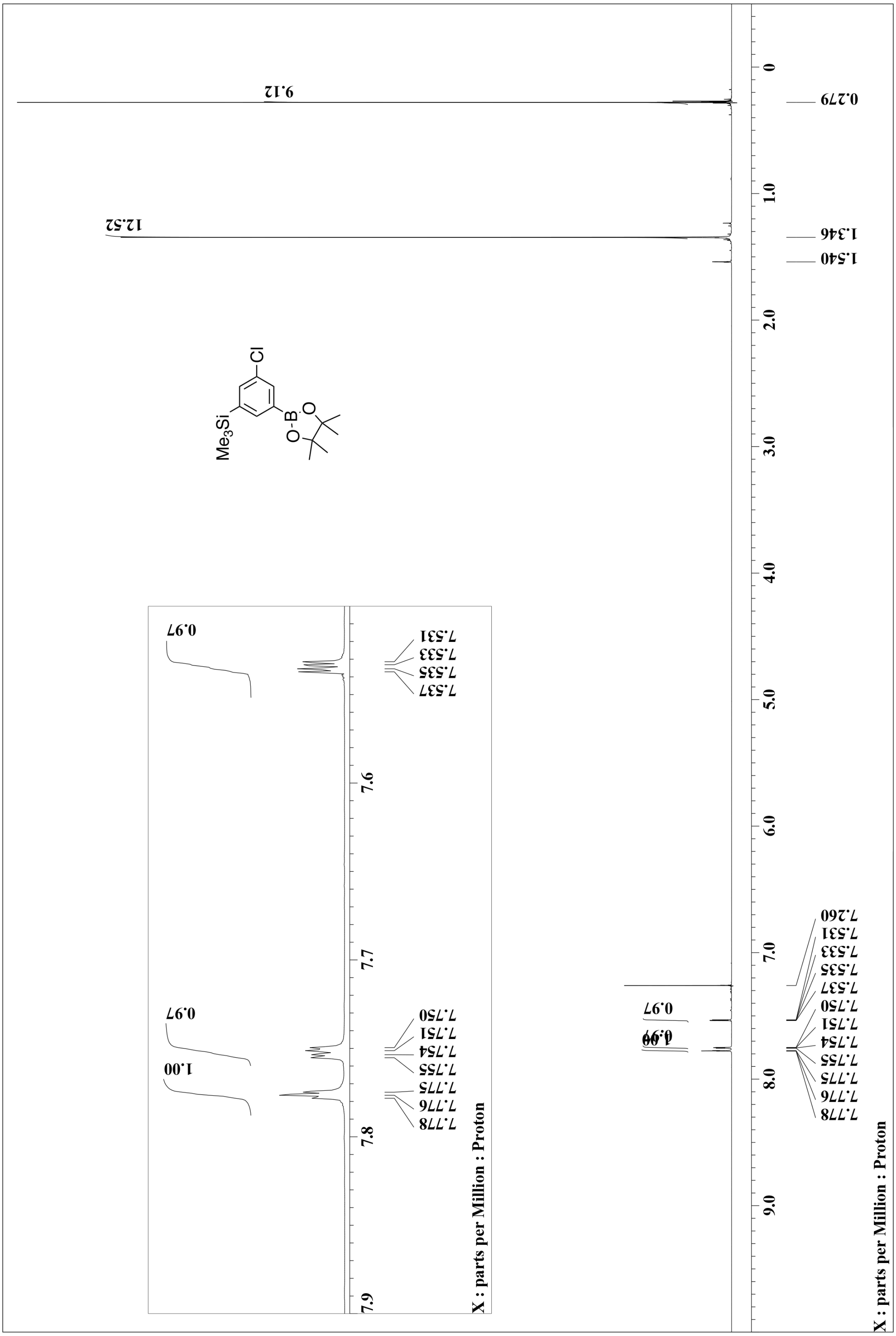

Supplementary Fig. 11 | ^{1}H NMR spectrum of 3 in $CDCl_3$.

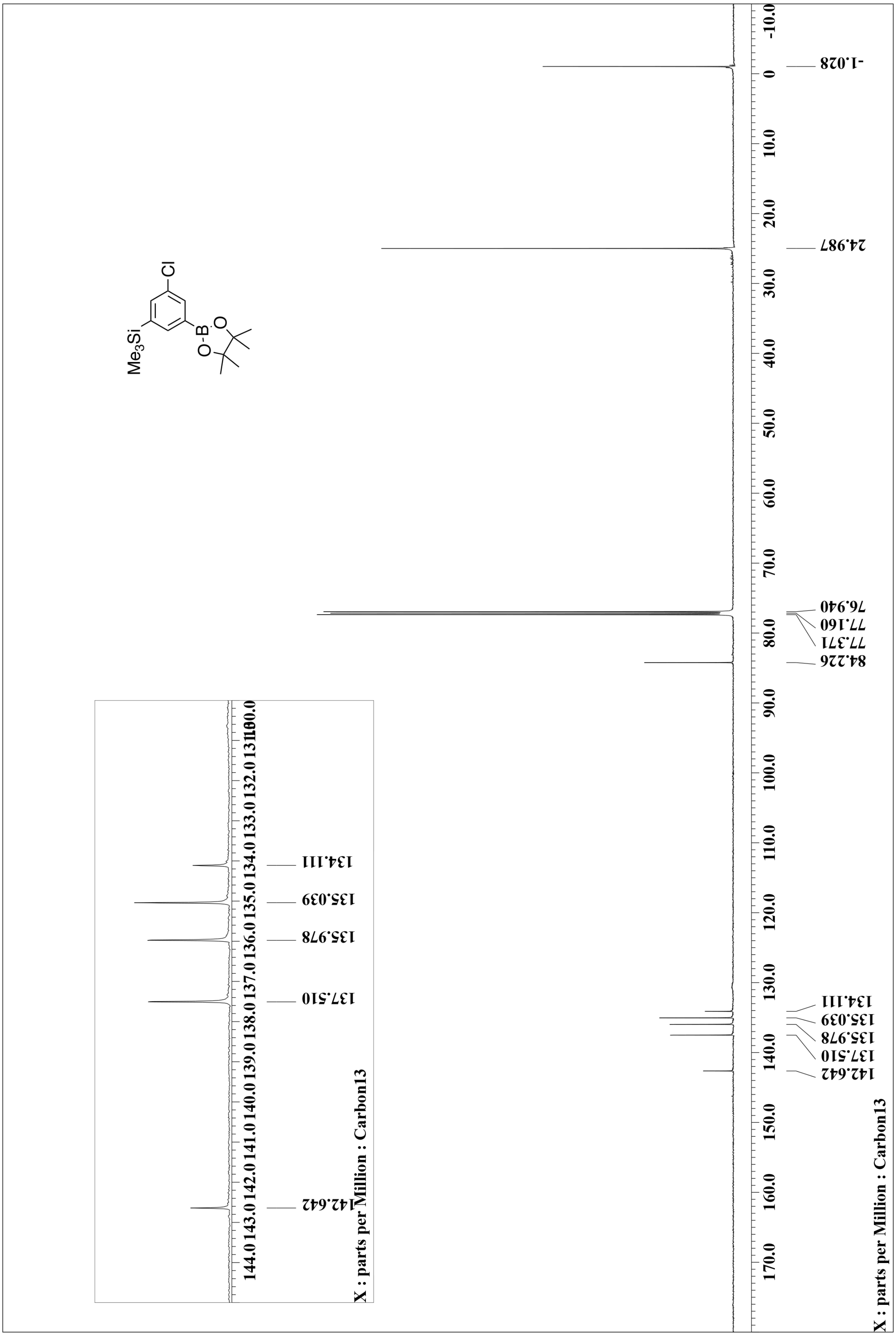

Supplementary Fig. 12 | ^{13}C NMR spectrum of 3 in $CDCl_3$.

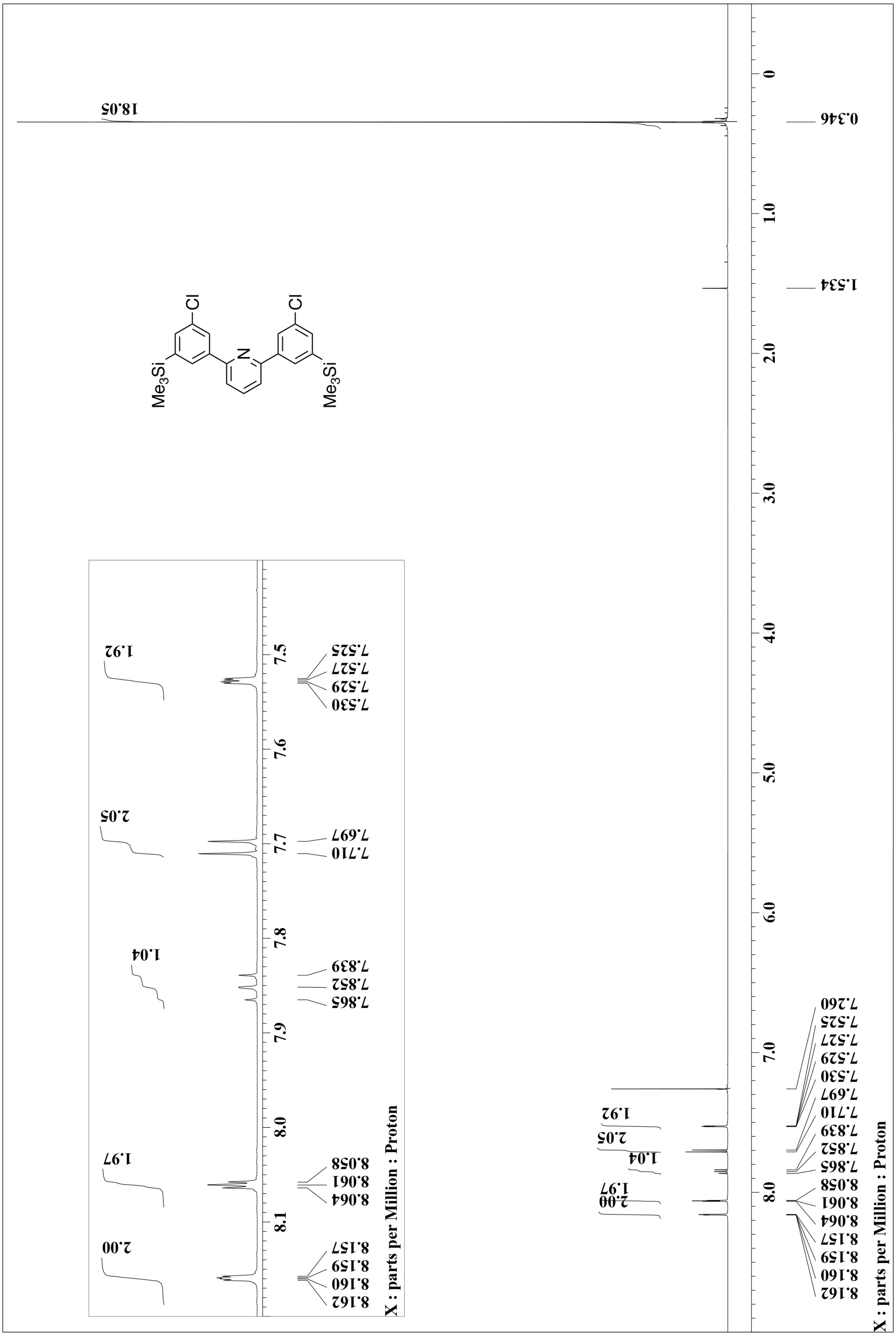

Supplementary Fig. 13 | ^{1}H NMR spectrum of 5 in $CDCl_3$.

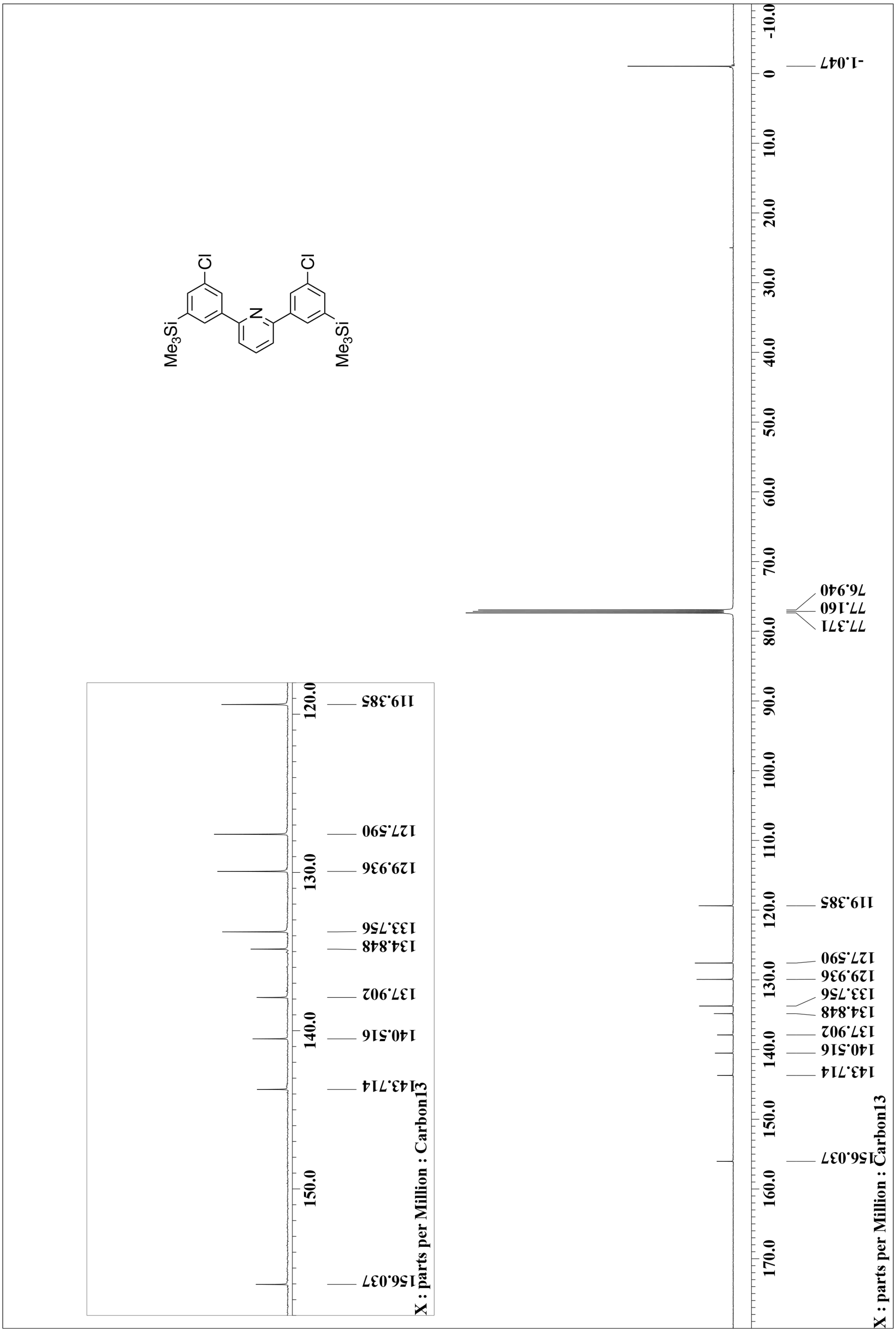

Supplementary Fig. 14 | ^{13}C NMR spectrum of 5 in $CDCl_3$.

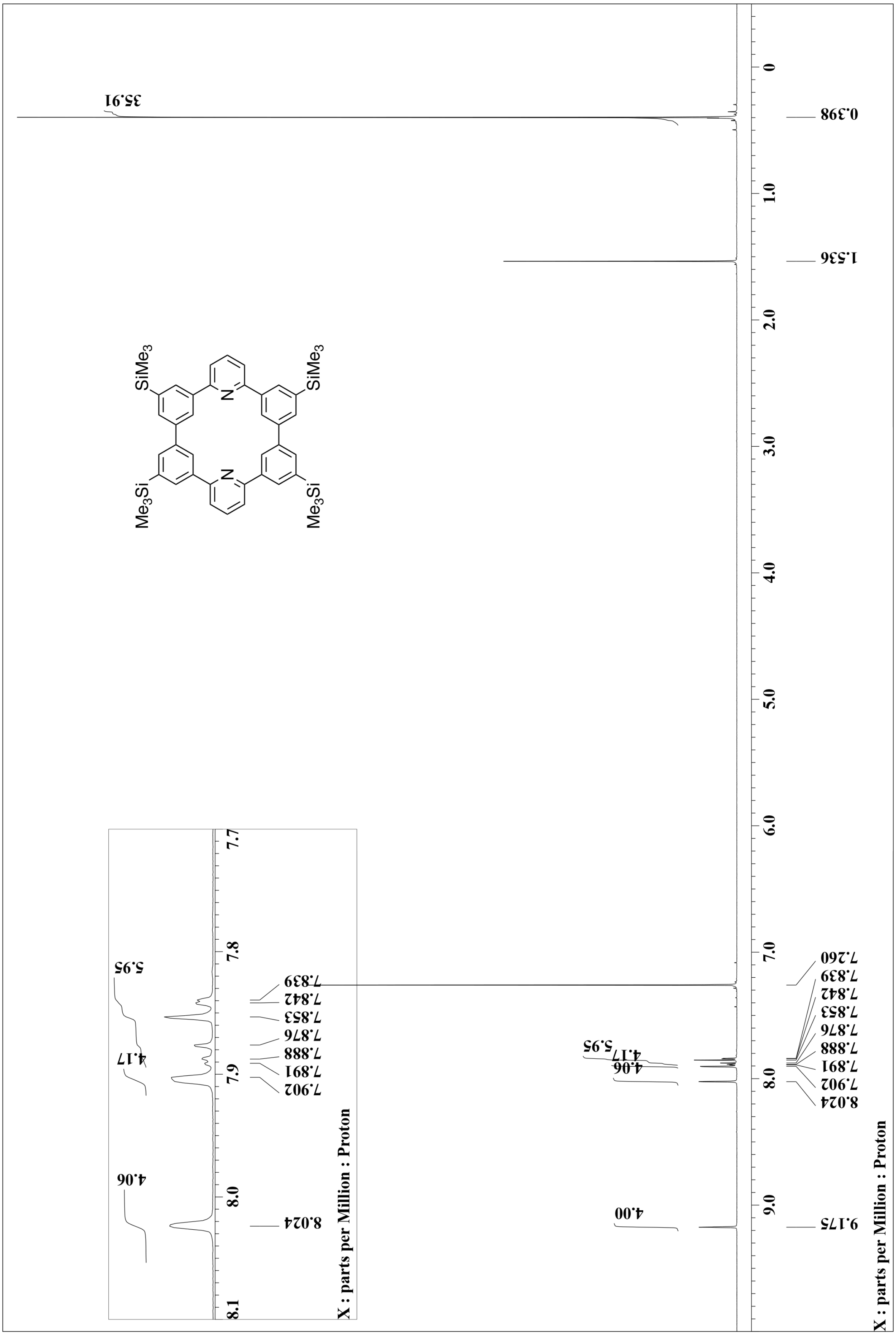

Supplementary Fig. 15 | ^{1}H NMR spectrum of 6 in $CDCl_3$.

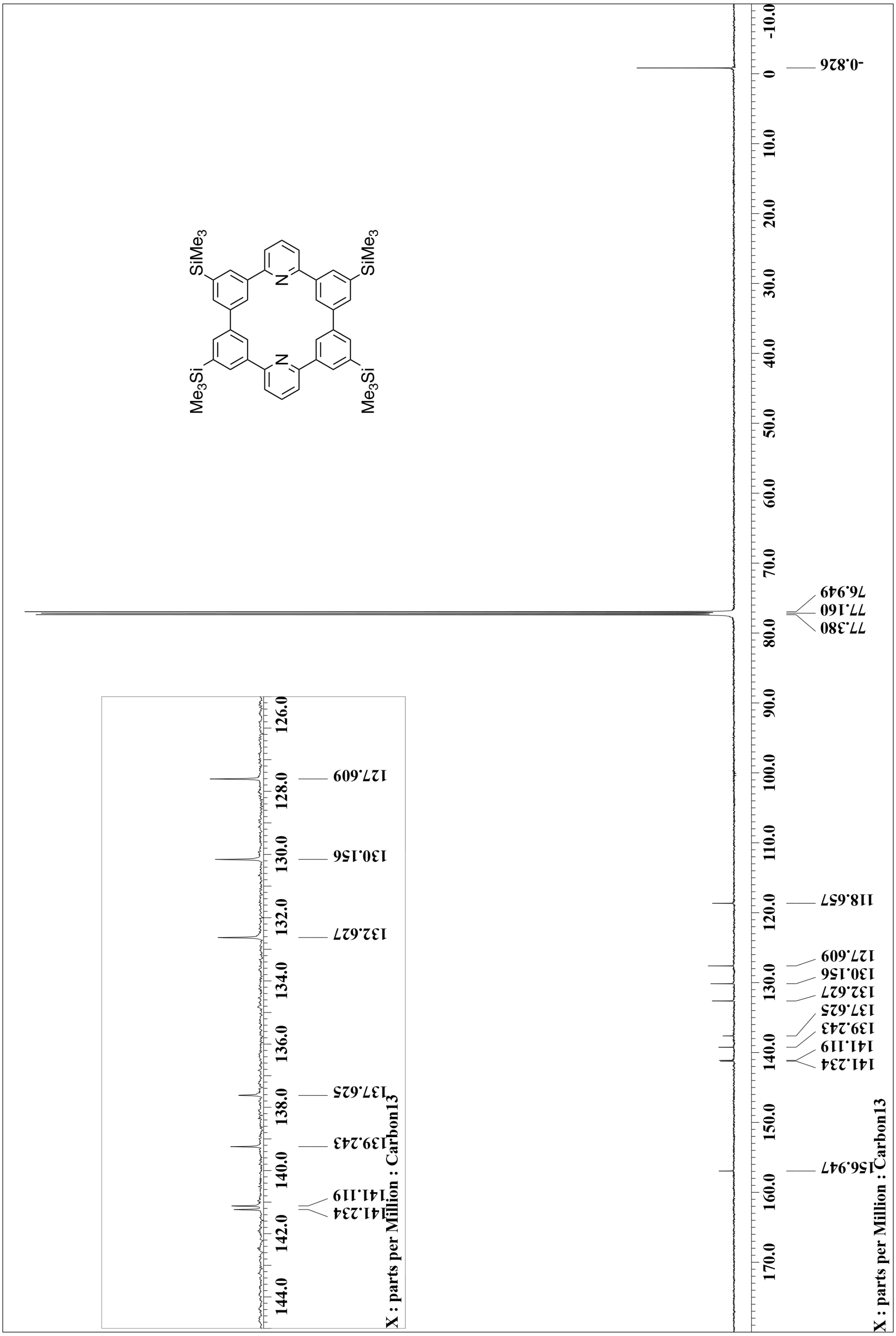

Supplementary Fig. 16 | ^{13}C NMR spectrum of 6 in $CDCl_3$.

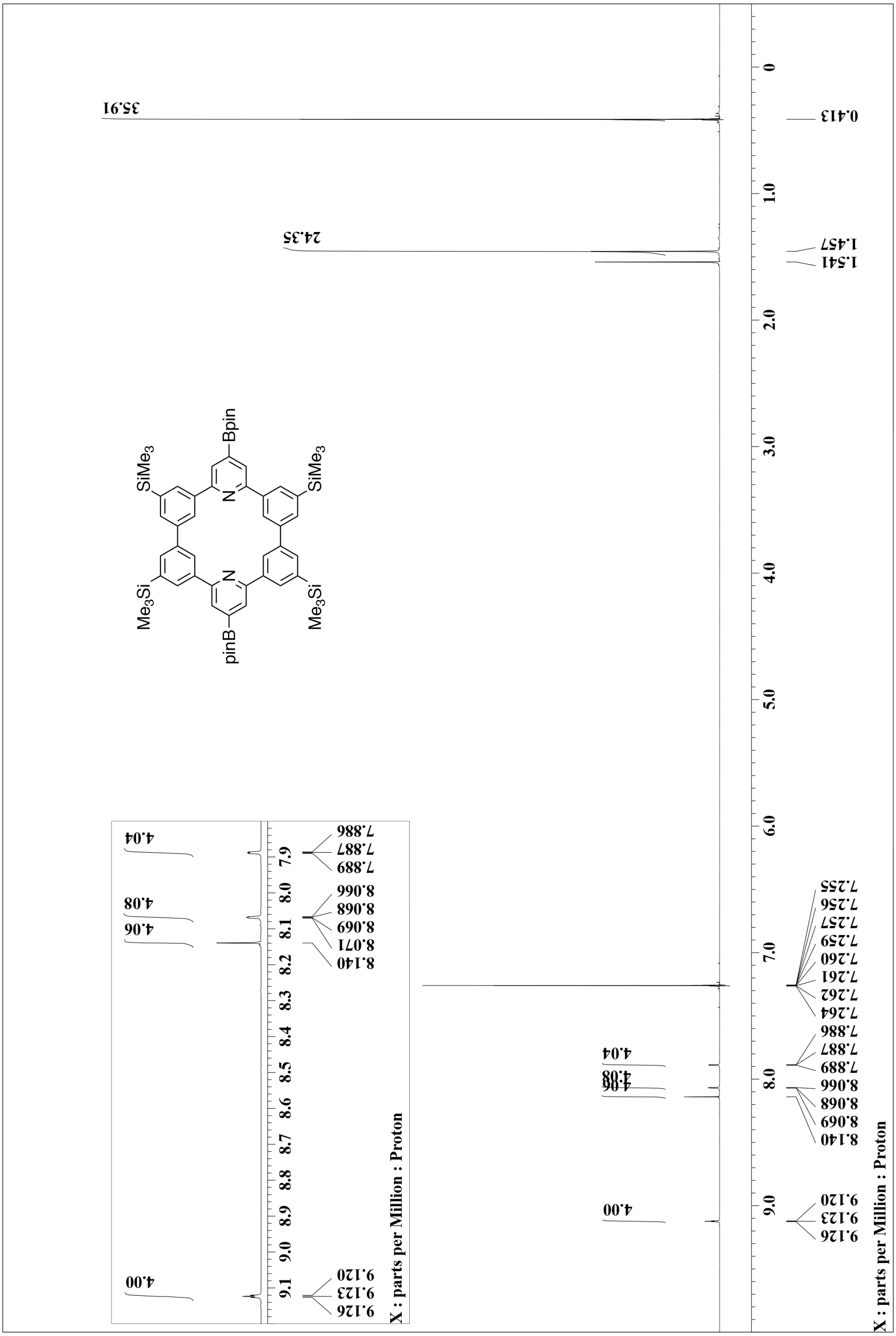

Supplementary Fig. 17 | ^{1}H NMR spectrum of 7 in $CDCl_3$.

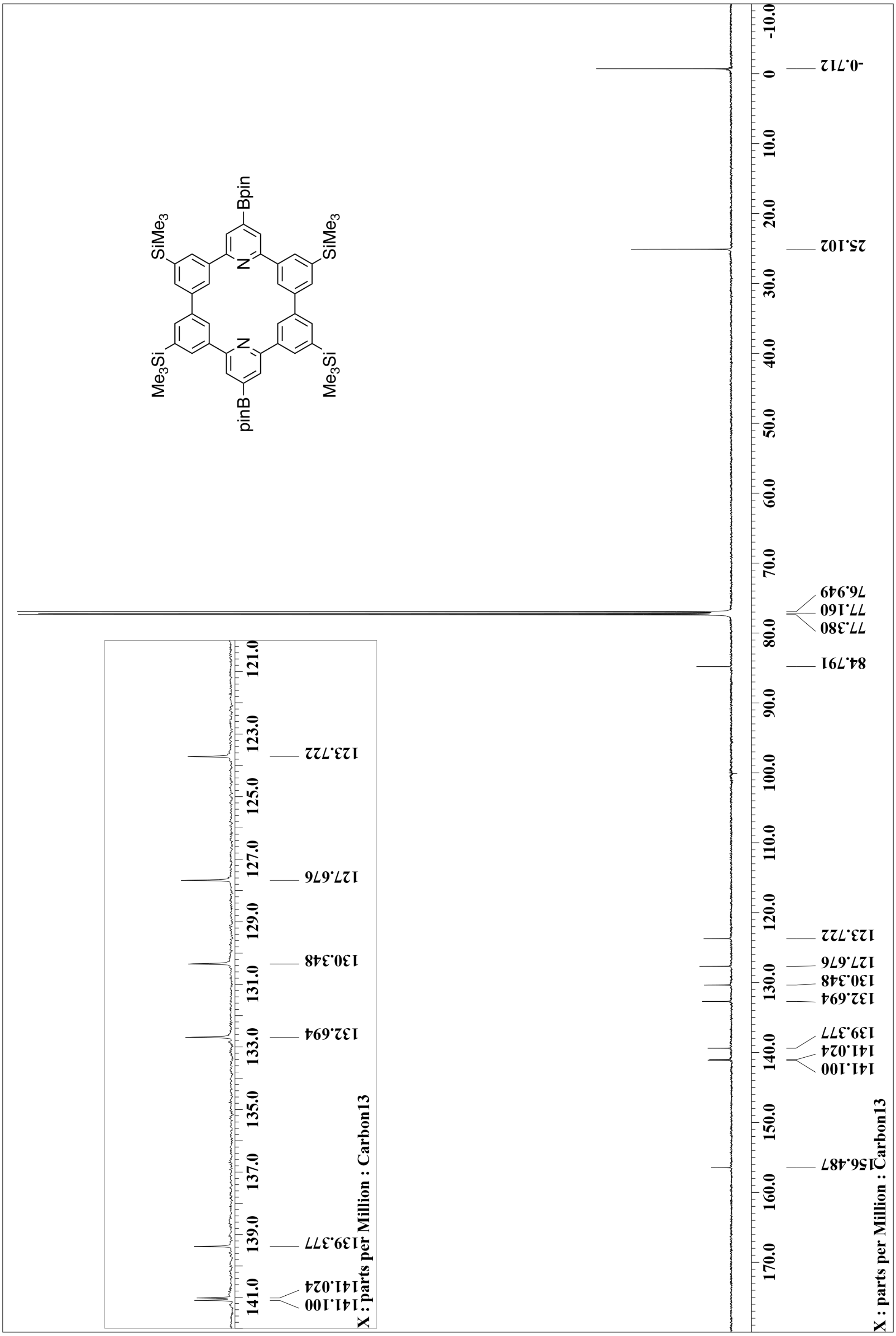

Supplementary Fig. 18 | ^{13}C NMR spectrum of 7 in $CDCl_3$.

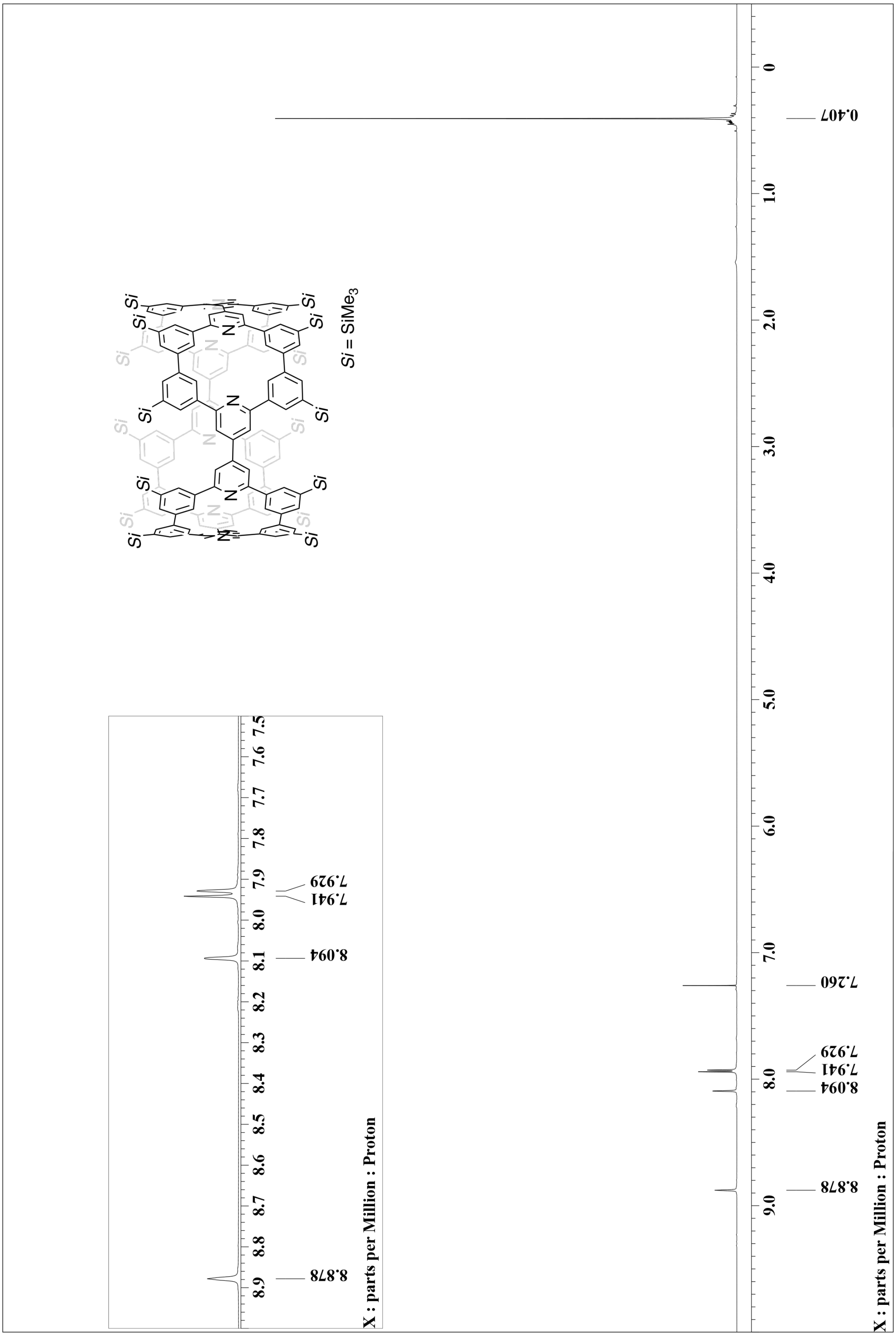

Supplementary Fig. 19 | ^{1}H NMR spectrum of 8 in $CDCl_3$.

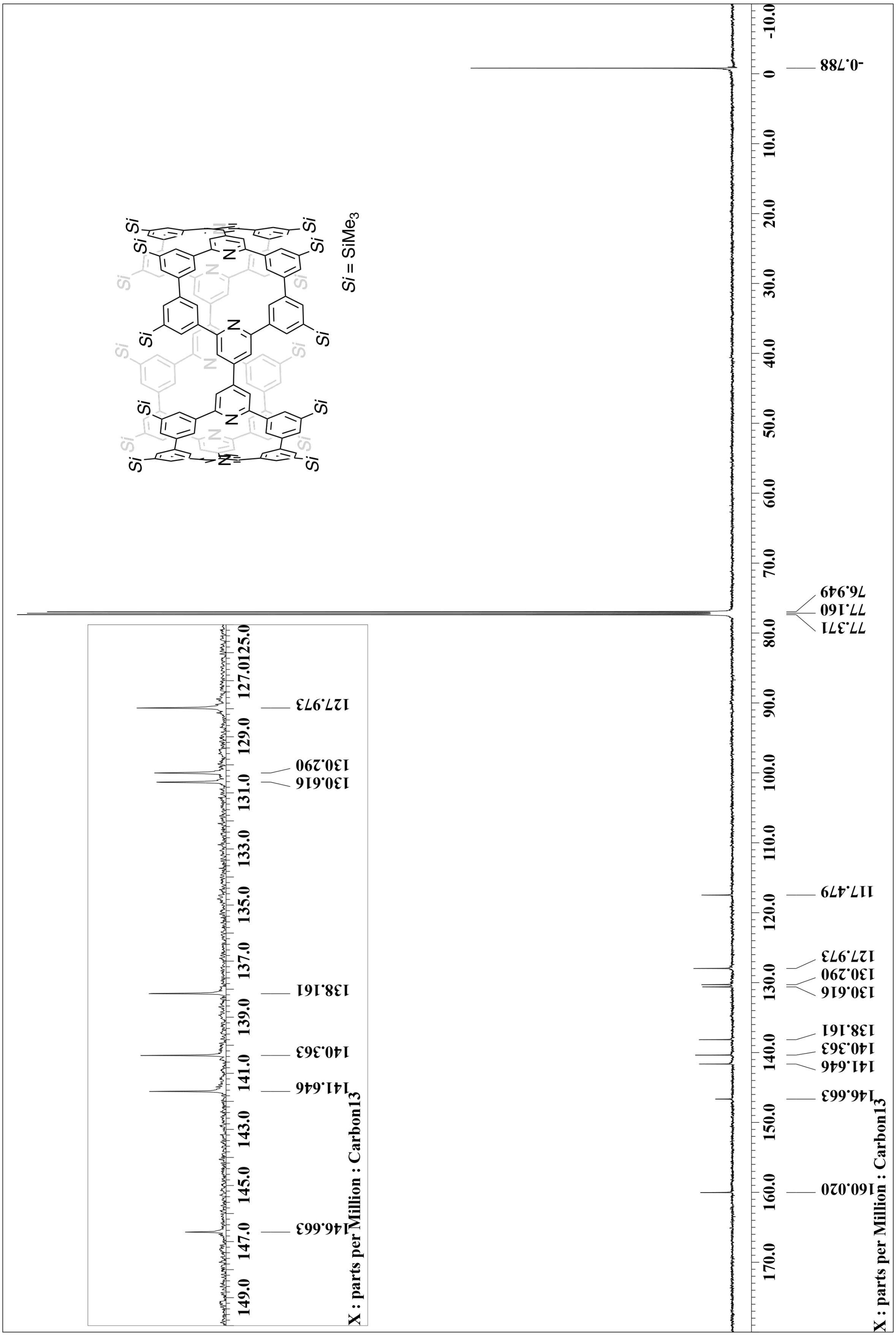

Supplementary Fig. 20 | ^{13}C NMR spectrum of 8 in $CDCl_3$.

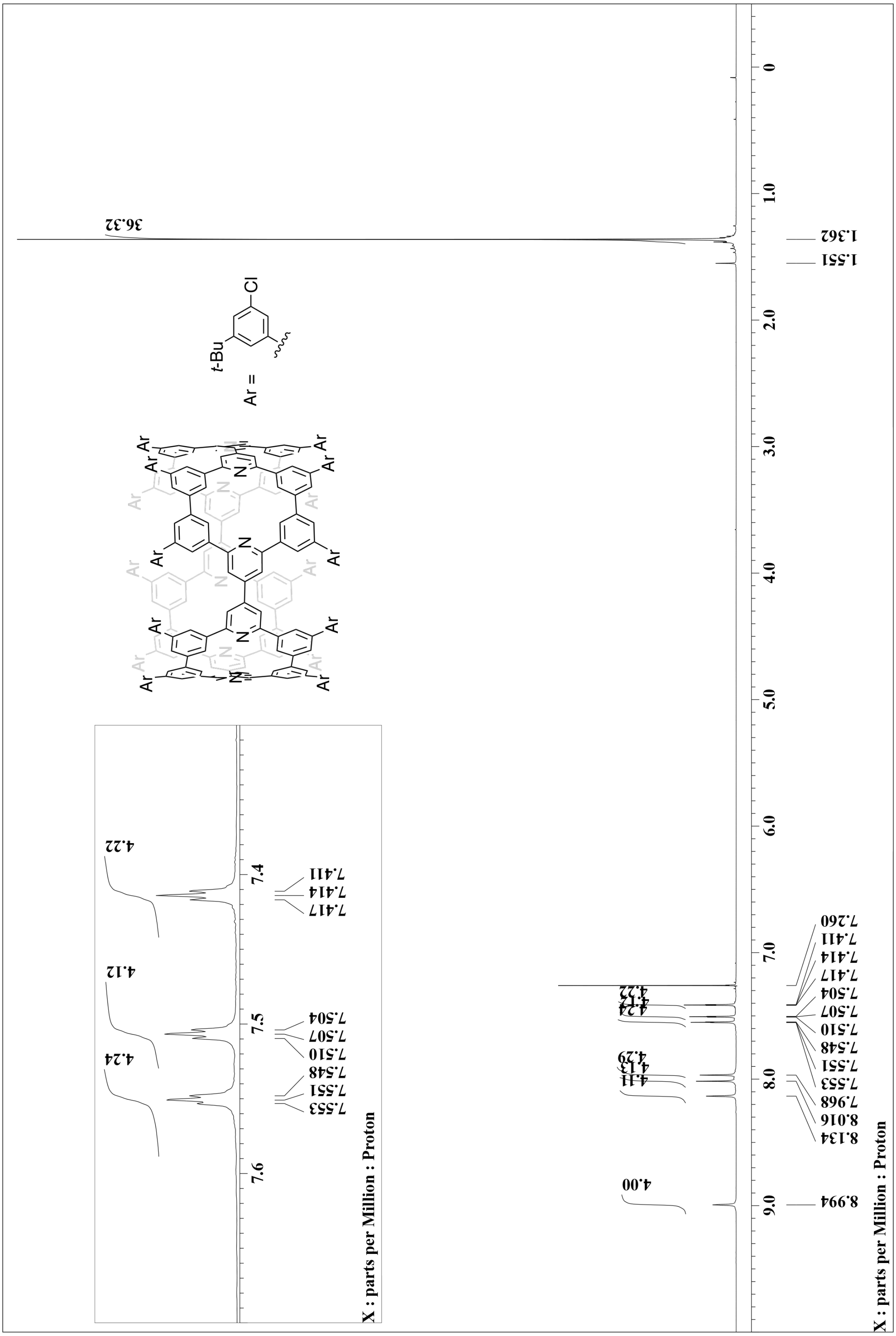

Supplementary Fig. 21 | ^{1}H NMR spectrum of 9 in $CDCl_3$.

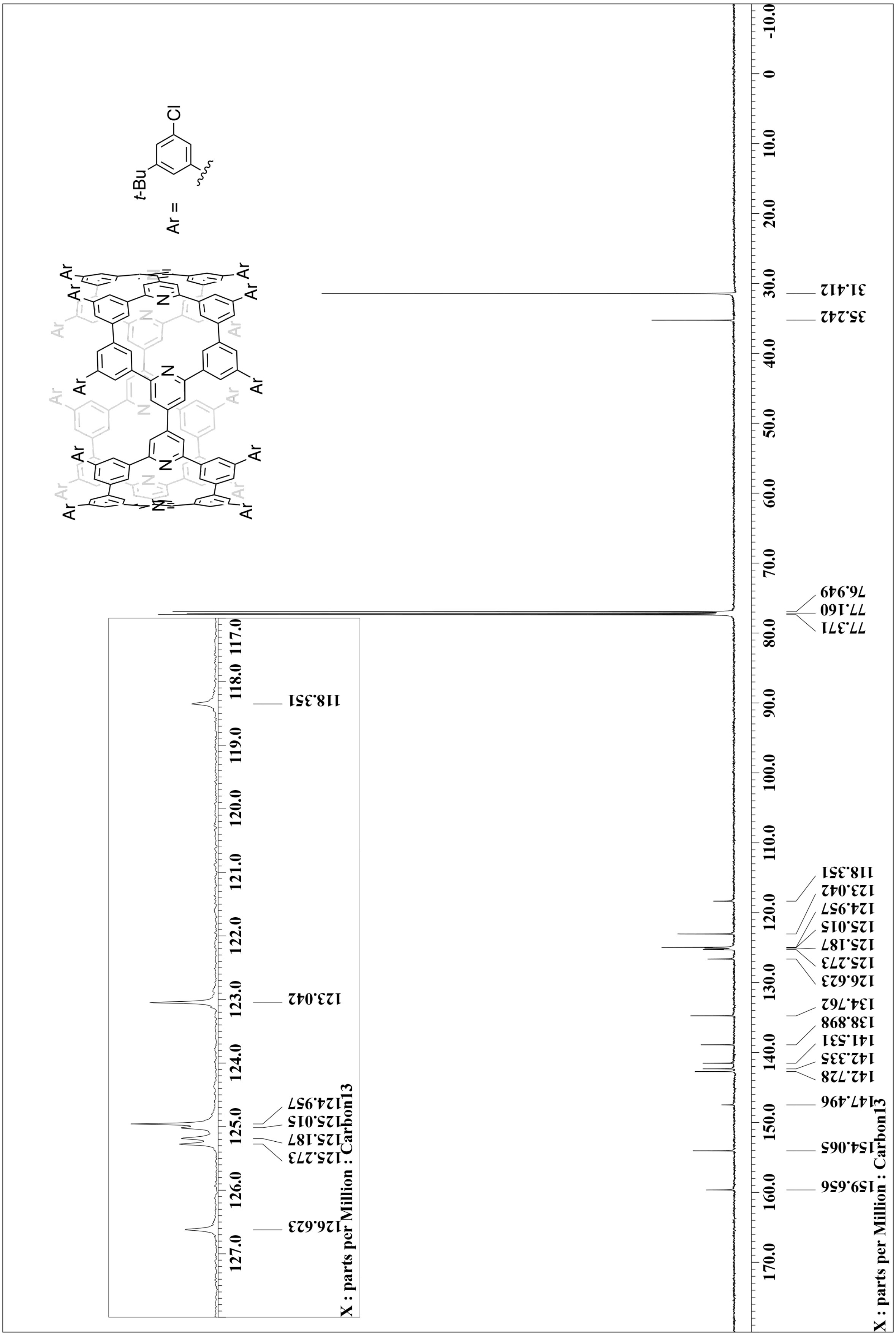

Supplementary Fig. 22 | ^{13}C NMR spectrum of 9 in $CDCl_3$.

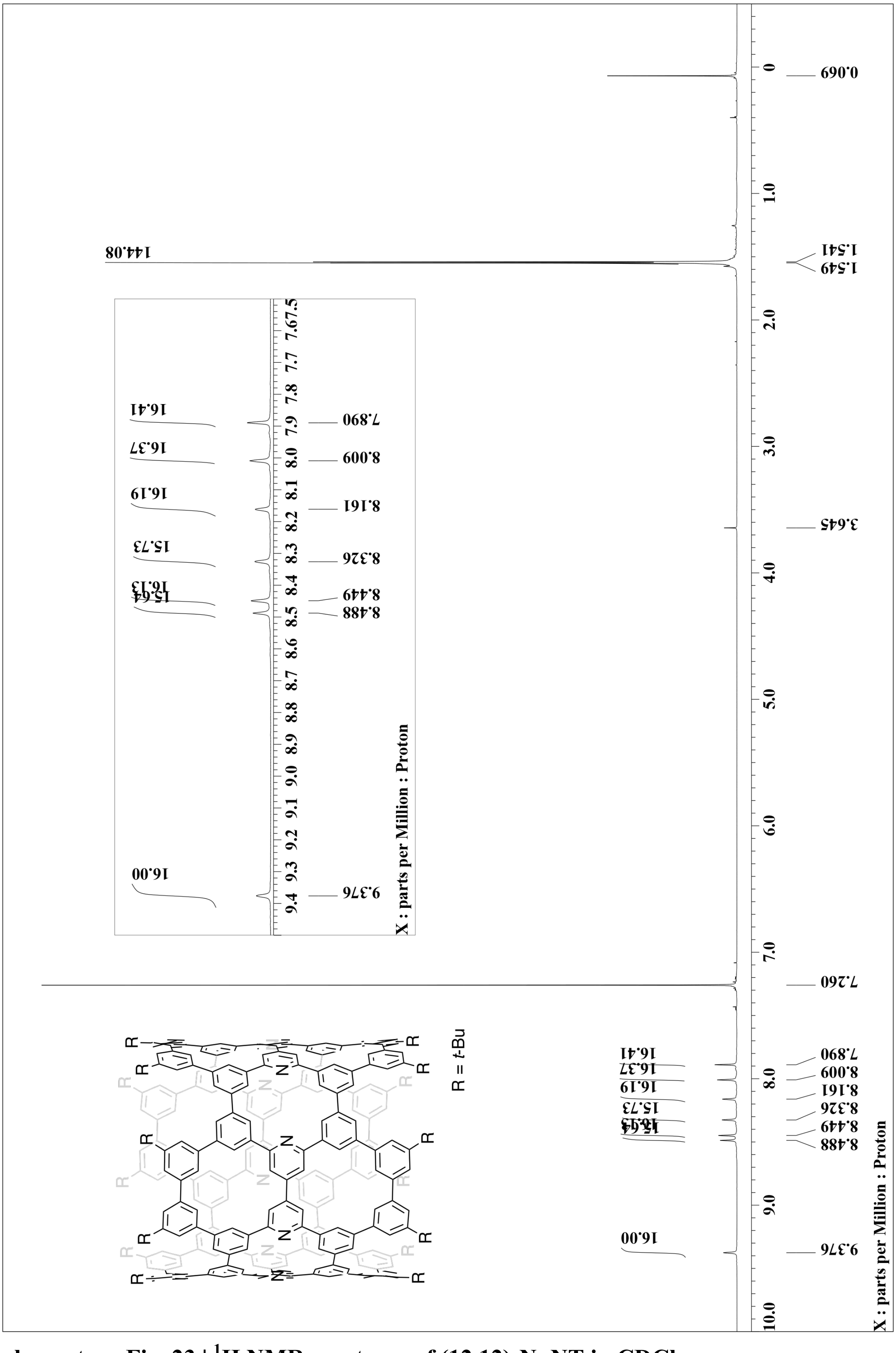

Supplementary Fig. 23 | ¹H NMR spectrum of (12,12)-NpNT in $CDCl_3$.

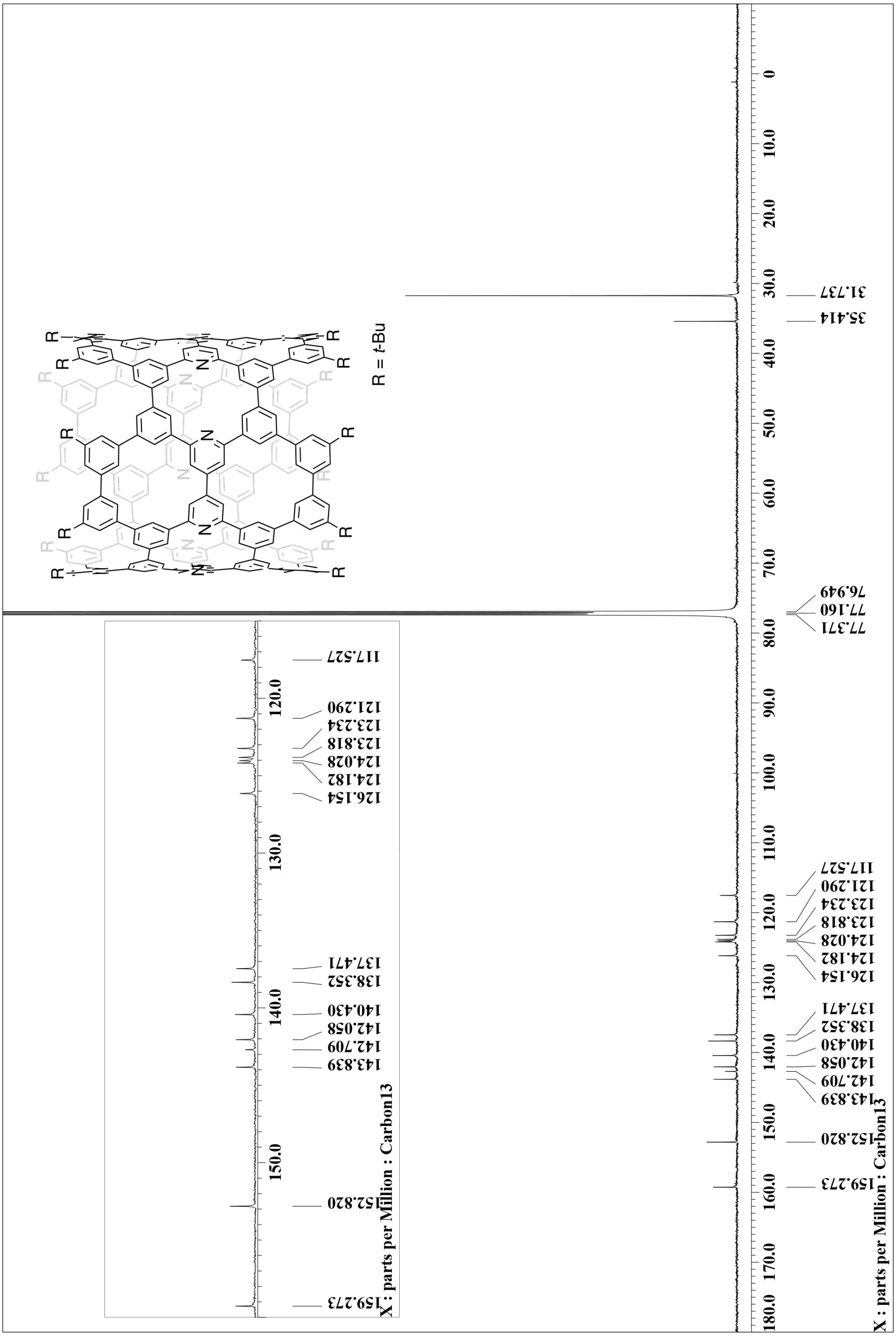

Supplementary Fig. 24 | ^{13}C NMR spectrum of (12,12)-NpNT in $CDCl_3$.

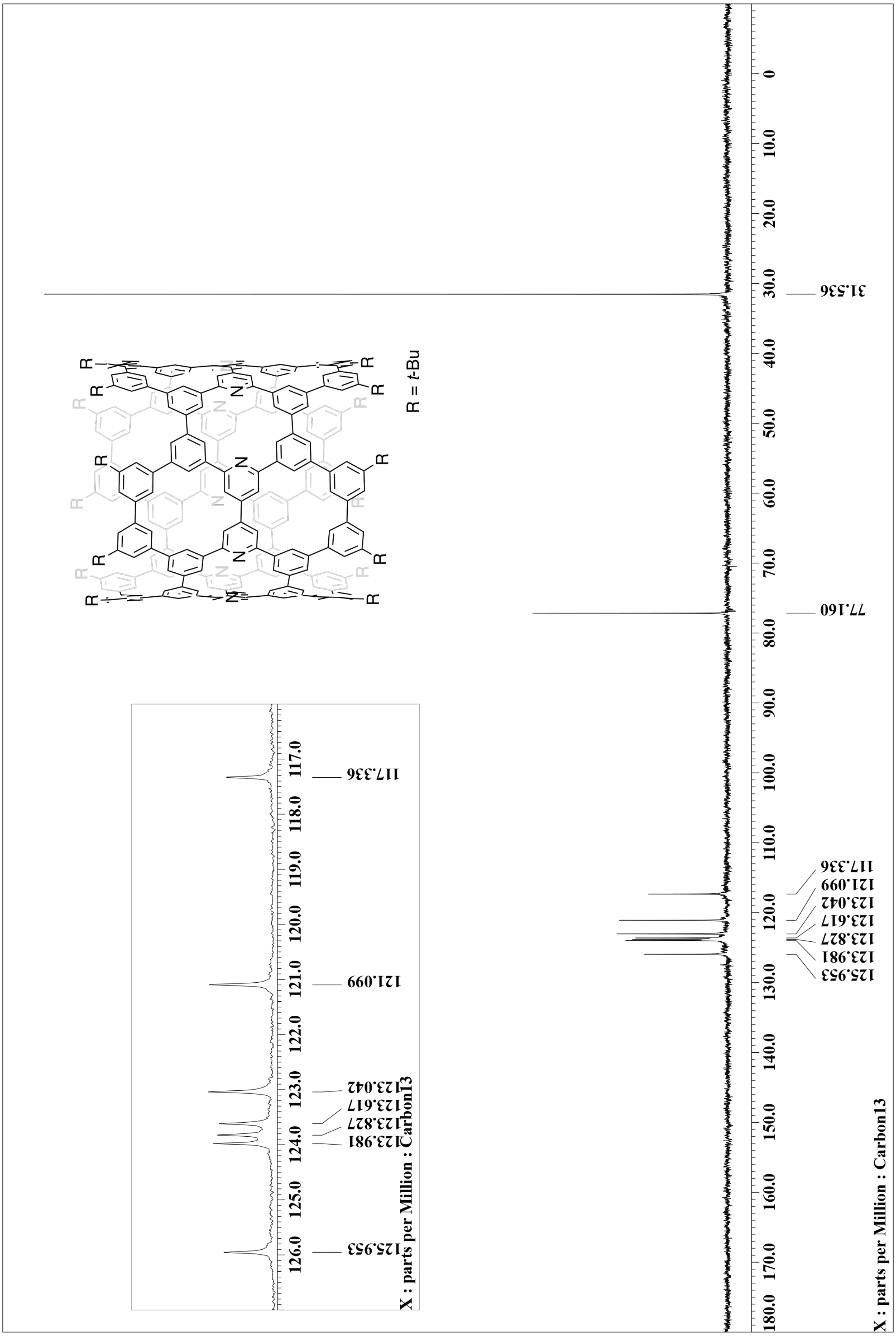

Supplementary Fig. 25 | DEPT135 NMR spectrum of (12,12)-NpNT in $CDCl_3$.

Supplementary References

1. Pangborn, A. B., Giardello, M. A., Grubbs, R. H., Rosen, R. K. & Timmers, F. J. Safe and convenient procedure for solvent purification. *Organometallics* **15**, 1518-1520 (1996).
2. Ikemoto, K., Kobayashi, R., Sato, S. & Isobe, H. Synthesis and bowl-in-bowl assembly of a geodesic phenylene bowl. *Angew. Chem. Int. Ed.* **56**, 6511-6514 (2017).
3. Haubold, W., Herdtle, J., Gollinger, W. & Einholz, W. Darstellung von arylhalogenboranen. *J. Organomet. Chem.* **315**, 1-8 (1986).
4. Saito, R., Dresselhaus, G., & Dresselhaus, M. S. *Physical Properties of Carbon Nanotubes* (Imperial College Press, London, 1998).
5. Matsuno, T., Naito, H., Hitosugi, S., Sato, S., Kotani, M. & Isobe H. Geometric measures of finite carbon nanotube molecules: a proposal for length index and filling indexes. *Pure Appl. Chem.* **86**, 489-495 (2014).
6. For F_b value, non-C-C bonds (eg. C-N) are not included, and for Fa value, non-carbon atoms (eg. N and H) are not included.
7. As is the case of the previous descriptor, the edge atoms must be included in a hexagon. Note also that the origin was not important for the previous indices (F_b, F_a and t_f) and was not clearly defined.
8. If multiple edge atoms are located at an identical distance from the atom with the highest priority, an atom with the second priority should be used as the second reference. An edge atom with the shortest distance from the second referene is assigned as the origin.
9. In (12,12)-NpNT molecule, there are multiple candidates of the origin from the present rule, and the choice makes no difference, as all of them result in identical coordinates.
10. The previous version can also be found at https://www.chem.s.u-tokyo.ac.jp/users/physorg/finite/
11. Sheldrick, G. M. *SHELXT* - integrated space-group and crystal-structure determination. *Acta Crystallogr. A* **71**, 3-8 (2015).
12. Sheldrick, G. M. A short history of SHELX. *Acta Crystallogr. A* **64**, 112-122 (2008).
13. Kabuto, C., Akine, S., Nemoto, T. & Kwon, E. Release of software (Yadokari-XG 2009) for crystal structure analyses. *J. Cryst. Soc. Jpn.* **51**, 218-224 (2009).
14. van der Sluis, P. & Spek, A. L. BYPASS: An effective method for the refinement of crystal structures containing disordered solvent regions. *Acta Crystallogr. A* **46**, 194-201 (1990).
15. Spek, A. L. Single-crystal structure validation with the program PLATON. *J. Appl. Cryst.* **36**, 7-13 (2003).
16. Brock, C. P., Dunitz, J. D. & Hirshfeld, F. L. Transferability of deformation densities among related molecules: Atomic multipole parameters from perylene for improved estimation of molecular vibrations in naphthalene and anthracene. *Acta Crystallogr. B* **47**, 789-797 (1991).
17. Volkov, A., Macchi, P., Farrugia, L. J., Gatti, C., Mallinson, P., Rihter, T. & Koritsánszky, T. *XD2016, a computer program package for multipole refinement, topological analysis of charge densities and evaluation of intermolecular energies from experimental and theoretical structure factors* (University at Buffalo; State University of New York; University of Milano; University of Glasgow; CNR-ISTM; Middle Tennessee State University, 2016).
18. Koritsanszky, T., Volkov, A. & Coppens, P. Aspherical-atom scattering factors from molecular wave functions. 1. Transferability and conformation dependence of atomic electron densities of peptides within the multipole formalism. *Acta Crystgallogr. A* **58**, 464-472 (2002).
19. Dominiak, P. M., Volkov, A., Li, X., Messerschmidt, M. & Coppens, P. A theoretical databank of transferable aspherical atoms and its application to electrostatic interaction energy calculations of macromolecules. *J. Chem. Theory Comput.* **3**, 232-247 (2007).
20. Sun, Z., Ikemoto, K., Fukunaga, T. M., Koretsune, T., Arita, R., Sato, S. & Isobe, H. Finite phenine nanotubes with periodic vacancy defects. *Science* **363**, 151-155 (2019).
21. Macrae, C. F., Bruno, I. J., Chisholm, J. A., Edgington, P. R., McCabe, P. Pidcock, E., Rodriguez-Monge, L., Taylor, R., van de Streek, J. & Wood, P. A. *Mercury CSD 2.0* – new features for the visualization and investigation of crystal structures. *J. Appl. Cryst.* **41**, 466-470 (2008).
22. Pettersen, E. F., Goddard, T. D., Huang, C. C., Couch, G. S., Greenblatt, D. M., Meng, E. C. & Ferrin, T. E. UCSF Chimera–a visualization system for exploratory research and analysis. *J. Comput. Chem.* **13**, 1605-1612 (2004).
23. Hübschle, C. B. & Dittrich, B. *MoleCoolQt* – a molecule viewer for charge-density research. *J. Appl. Cryst.* **44**, 238-240 (2011).
24. Hübschle, C. B. & Luger, P. *MolIso* – a program for colour-mapped iso-surfaces. *J. Appl. Cryst.* **39**, 901-904 (2006).
25. CrystalClear-SM Expert 2.1 b29 (Rigaku Corporation, Tokyo, Japan, 2013).

26. CrysAlisPro version 1.171.39.46 (Rigaku Corporation, Tokyo, Japan, 2018).
27. Näther, C., Riedel, J. & Jeß, I. 4,4'-Bipyridine dihydrate at 130 K. *Acta Crystallogr. C* **57**, 111-112 (2001).
28. Perdew, J. P., Burke, K. & Ernzerhof, M. Generalized gradient approximation made simple. *Phys. Rev. Lett.* **77**, 3865-3868 (1996).
29. Hehre, W. J., Stewart, R. F. & Pople, J. A. Self-consistent molecular-orbital methods. I. Use of Gaussian expansions of Slater-type atomic orbitals. *J. Chem. Phys.* **51**, 2657-2664 (1969).
30. Frisch, M. J., Trucks, G. W., Schlegel, H. B., Scuseria, G. E., Robb, M. A. Cheeseman, J. R., Scalmani, G., Barone, V., Petersson, G. A., Nakatsuji, H., Li, X., Caricato, M., Marenich, A. V., Bloino, J., Janesko, B. G., Gomperts, R., Mennucci, B., Hratchian, H. P., Ortiz, J. V., Izmaylov, A. F., Sonnenberg, J. L., Williams-Young, D., Ding, F., Lipparini, F., Egidi, F., Goings, J., Peng, B., Petrone, A., Henderson, T., Ranasinghe, D., Zakrzewski, V. G., Gao, J., Rega, N., Zheng, G., Liang, W., Hada, M., Ehara, M., Toyota, K., Fukuda, R., Hasegawa, J., Ishida, M., Nakajima, T., Honda, Y., Kitao, O., Nakai, H., Vreven, T., Throssell, K., Montgomery, Jr. J. A., Peralta, J. E., Ogliaro, F., Bearpark, M. J., Heyd, J. J., Brothers. E. N., Kudin, K. N., Staroverov, V. N., Keith, T. A., Kobayashi, R., Normand, J., Raghavachari, K., Rendell, A. P., Burant, J. C., Iyengar, S. S., Tomasi, J., Cossi, M., Millam, J. M., Klene, M., Adamo, C., Cammi, R., Ochterski, J. W., Martin, R. L., Morokuma, K., Farkas, O., Foresman, J. B. & Fox, D. J. *Gaussian 16, Revision B.01*, (Gaussian, Inc., Wallingford CT, 2016).
