# Supplementary material for: A nitrogen-doped nanotube molecule with atom vacancy defects
Source: Nat Commun. 2020 Apr 14;11:1807. doi: 10.1038/s41467-020-15662-6 (PMC7156684; doi:10.1038/s41467-020-15662-6)
Supplement: Supplementary file 3 — Description of Additional Supplementary Files [file 41467_2020_15662_MOESM3_ESM.pdf]

Description of Additional Supplementary Files

File Name: Supplementary Data 1
Description: Cartesian coordinates of (12,12)-NpNT

File Name: Supplementary Data 2
Description: Cartesian coordinates of 4,4'-bipyridine
