# Supplementary material for: A nitrogen-doped nanotube molecule with atom vacancy defects
Source: Nat Commun. 2020 Apr 14;11:1807. doi: 10.1038/s41467-020-15662-6 (PMC7156684; doi:10.1038/s41467-020-15662-6)
Supplement: Supplementary file 4 — Supplementary Data 1 [file 41467_2020_15662_MOESM4_ESM.doc]

**Supplementary Data 1 | Cartesian coordinates of (12,12)-NpNT.**

---------------------------------------------------------------------

Center Atomic Atomic Coordinates (Angstroms)

Number Number Type X Y Z

---------------------------------------------------------------------

1 6 0 -3.626189 8.413664 7.446112

2 6 0 -2.932334 8.756459 8.635688

3 6 0 -2.912576 8.126374 6.251599

4 6 0 -1.514794 8.793981 8.628608

5 6 0 -1.491846 8.174041 6.276082

6 6 0 -0.760203 8.497567 7.454217

7 6 0 0.760203 8.497567 7.454217

8 6 0 1.514794 8.793981 8.628608

9 6 0 1.491846 8.174041 6.276082

10 6 0 2.932334 8.756459 8.635688

11 6 0 2.912576 8.126374 6.251599

12 6 0 3.626189 8.413664 7.446112

13 6 0 8.413664 3.626189 7.446112

14 6 0 8.756459 2.932334 8.635688

15 6 0 8.126374 2.912576 6.251599

16 6 0 8.793981 1.514794 8.628608

17 6 0 8.174041 1.491846 6.276082

18 6 0 8.497567 0.760203 7.454217

19 6 0 8.497567 -0.760203 7.454217

20 6 0 8.793981 -1.514794 8.628608

21 6 0 8.174041 -1.491846 6.276082

22 6 0 8.756459 -2.932334 8.635688

23 6 0 8.126374 -2.912576 6.251599

24 6 0 8.413664 -3.626189 7.446112

25 6 0 3.626189 -8.413664 7.446112

26 6 0 2.932334 -8.756459 8.635688

27 6 0 2.912576 -8.126374 6.251599

28 6 0 1.514794 -8.793981 8.628608

29 6 0 1.491846 -8.174041 6.276082

30 6 0 0.760203 -8.497567 7.454217

31 6 0 -0.760203 -8.497567 7.454217

32 6 0 -1.514794 -8.793981 8.628608

33 6 0 -1.491846 -8.174041 6.276082

34 6 0 -2.932334 -8.756459 8.635688

35 6 0 -2.912576 -8.126374 6.251599

36 6 0 -3.626189 -8.413664 7.446112

37 6 0 -8.413664 -3.626189 7.446112

38 6 0 -8.756459 -2.932334 8.635688

39 6 0 -8.126374 -2.912576 6.251599

40 6 0 -8.793981 -1.514794 8.628608

41 6 0 -8.174041 -1.491846 6.276082

42 6 0 -8.497567 -0.760203 7.454217

43 6 0 -8.497567 0.760203 7.454217

44 6 0 -8.793981 1.514794 8.628608

45 6 0 -8.174041 1.491846 6.276082

46 6 0 -8.756459 2.932334 8.635688

47 6 0 -8.126374 2.912576 6.251599

48 6 0 -8.413664 3.626189 7.446112

49 1 0 0.994780 -9.051416 9.566255

50 1 0 0.952317 -7.916588 5.351699

51 1 0 -0.952317 -7.916588 5.351699

52 1 0 -0.994780 -9.051416 9.566255

53 1 0 -4.729850 -8.382767 7.446781

54 1 0 -8.382767 -4.729850 7.446781

55 1 0 -9.051416 -0.994780 9.566255

56 1 0 -9.051416 0.994780 9.566255

57 1 0 -7.916588 -0.952317 5.351699

58 1 0 -7.916588 0.952317 5.351699

59 1 0 -8.382767 4.729850 7.446781

60 1 0 -4.729850 8.382767 7.446781

61 1 0 -0.952317 7.916588 5.351699

62 1 0 -0.994780 9.051416 9.566255

63 1 0 0.994780 9.051416 9.566255

64 1 0 0.952317 7.916588 5.351699

65 1 0 4.729850 8.382767 7.446781

66 1 0 8.382767 4.729850 7.446781

67 1 0 9.051416 0.994780 9.566255

68 1 0 7.916588 0.952317 5.351699

69 1 0 7.916588 -0.952317 5.351699

70 1 0 9.051416 -0.994780 9.566255

71 1 0 8.382767 -4.729850 7.446781

72 1 0 4.729850 -8.382767 7.446781

73 6 0 -4.724810 6.851502 4.957174

74 6 0 -4.727015 6.827552 -2.513774

75 6 0 -3.608122 7.732127 4.963346

76 6 0 -3.605779 7.697625 -2.491514

77 6 0 -3.067813 8.160028 3.721996

78 6 0 -3.067813 8.160028 -3.721996

79 6 0 3.067813 8.160028 3.721996

80 6 0 3.067813 8.160028 -3.721996

81 6 0 3.608122 7.732127 4.963346

82 6 0 3.605779 7.697625 -2.491514

83 6 0 4.724810 6.851502 4.957174

84 6 0 4.727015 6.827552 -2.513774

85 6 0 5.298090 6.373213 3.739455

86 6 0 5.298090 6.373213 -3.739455

87 6 0 6.373213 5.298090 3.739455

88 6 0 6.373213 5.298090 -3.739455

89 6 0 6.851502 4.724810 4.957174

90 6 0 6.827552 4.727015 -2.513774

91 6 0 7.732127 3.608122 4.963346

92 6 0 7.697625 3.605779 -2.491514

93 6 0 8.160028 3.067813 3.721996

94 6 0 8.160028 3.067813 -3.721996

95 6 0 8.160028 -3.067813 3.721996

96 6 0 8.160028 -3.067813 -3.721996

97 6 0 7.732127 -3.608122 4.963346

98 6 0 7.697625 -3.605779 -2.491514

99 6 0 6.851502 -4.724810 4.957174

100 6 0 6.827552 -4.727015 -2.513774

101 6 0 6.373213 -5.298090 3.739455

102 6 0 6.373213 -5.298090 -3.739455

103 6 0 5.298090 -6.373213 3.739455

104 6 0 5.298090 -6.373213 -3.739455

105 6 0 4.724810 -6.851502 4.957174

106 6 0 4.727015 -6.827552 -2.513774

107 6 0 3.608122 -7.732127 4.963346

108 6 0 3.605779 -7.697625 -2.491514

109 6 0 3.067813 -8.160028 3.721996

110 6 0 3.067813 -8.160028 -3.721996

111 6 0 -3.067813 -8.160028 3.721996

112 6 0 -3.067813 -8.160028 -3.721996

113 6 0 -3.608122 -7.732127 4.963346

114 6 0 -3.605779 -7.697625 -2.491514

115 6 0 -4.724810 -6.851502 4.957174

116 6 0 -4.727015 -6.827552 -2.513774

117 6 0 -5.298090 -6.373213 3.739455

118 6 0 -5.298090 -6.373213 -3.739455

119 6 0 -6.373213 -5.298090 3.739455

120 6 0 -6.373213 -5.298090 -3.739455

121 6 0 -6.851502 -4.724810 4.957174

122 6 0 -6.827552 -4.727015 -2.513774

123 6 0 -7.732127 -3.608122 4.963346

124 6 0 -7.697625 -3.605779 -2.491514

125 6 0 -8.160028 -3.067813 3.721996

126 6 0 -8.160028 -3.067813 -3.721996

127 6 0 -8.160028 3.067813 3.721996

128 6 0 -8.160028 3.067813 -3.721996

129 6 0 -7.732127 3.608122 4.963346

130 6 0 -7.697625 3.605779 -2.491514

131 6 0 -6.851502 4.724810 4.957174

132 6 0 -6.827552 4.727015 -2.513774

133 6 0 -6.373213 5.298090 3.739455

134 6 0 -6.373213 5.298090 -3.739455

135 6 0 -5.298090 6.373213 3.739455

136 6 0 -5.298090 6.373213 -3.739455

137 6 0 -4.727015 6.827552 2.513774

138 6 0 -4.724810 6.851502 -4.957174

139 6 0 -3.605779 7.697625 2.491514

140 6 0 -3.608122 7.732127 -4.963346

141 6 0 -3.626189 8.413664 -7.446112

142 6 0 -2.900191 7.978891 1.177120

143 6 0 -2.912576 8.126374 -6.251599

144 6 0 -2.900191 7.978891 -1.177120

145 6 0 -2.932334 8.756459 -8.635688

146 6 0 -1.500790 8.234091 1.203625

147 6 0 -1.491846 8.174041 -6.276082

148 6 0 -1.500790 8.234091 -1.203625

149 6 0 -1.514794 8.793981 -8.628608

150 6 0 -0.756099 8.328942 0.000000

151 6 0 -0.760203 8.497567 -7.454217

152 6 0 0.756099 8.328942 0.000000

153 6 0 0.760203 8.497567 -7.454217

154 6 0 1.500790 8.234091 1.203625

155 6 0 1.491846 8.174041 -6.276082

156 6 0 1.500790 8.234091 -1.203625

157 6 0 1.514794 8.793981 -8.628608

158 6 0 2.900191 7.978891 1.177120

159 6 0 2.912576 8.126374 -6.251599

160 6 0 2.900191 7.978891 -1.177120

161 6 0 2.932334 8.756459 -8.635688

162 6 0 3.605779 7.697625 2.491514

163 6 0 3.608122 7.732127 -4.963346

164 6 0 3.626189 8.413664 -7.446112

165 6 0 4.727015 6.827552 2.513774

166 6 0 4.724810 6.851502 -4.957174

167 6 0 6.827552 4.727015 2.513774

168 6 0 6.851502 4.724810 -4.957174

169 6 0 7.697625 3.605779 2.491514

170 6 0 7.732127 3.608122 -4.963346

171 6 0 8.413664 3.626189 -7.446112

172 6 0 7.978891 2.900191 1.177120

173 6 0 8.126374 2.912576 -6.251599

174 6 0 7.978891 2.900191 -1.177120

175 6 0 8.756459 2.932334 -8.635688

176 6 0 8.234091 1.500790 1.203625

177 6 0 8.174041 1.491846 -6.276082

178 6 0 8.234091 1.500790 -1.203625

179 6 0 8.793981 1.514794 -8.628608

180 6 0 8.328942 0.756099 0.000000

181 6 0 8.497567 0.760203 -7.454217

182 6 0 8.328942 -0.756099 0.000000

183 6 0 8.497567 -0.760203 -7.454217

184 6 0 8.234091 -1.500790 1.203625

185 6 0 8.174041 -1.491846 -6.276082

186 6 0 8.234091 -1.500790 -1.203625

187 6 0 8.793981 -1.514794 -8.628608

188 6 0 7.978891 -2.900191 1.177120

189 6 0 8.126374 -2.912576 -6.251599

190 6 0 7.978891 -2.900191 -1.177120

191 6 0 8.756459 -2.932334 -8.635688

192 6 0 7.697625 -3.605779 2.491514

193 6 0 7.732127 -3.608122 -4.963346

194 6 0 8.413664 -3.626189 -7.446112

195 6 0 6.827552 -4.727015 2.513774

196 6 0 6.851502 -4.724810 -4.957174

197 6 0 4.727015 -6.827552 2.513774

198 6 0 4.724810 -6.851502 -4.957174

199 6 0 3.605779 -7.697625 2.491514

200 6 0 3.608122 -7.732127 -4.963346

201 6 0 3.626189 -8.413664 -7.446112

202 6 0 2.900191 -7.978891 1.177120

203 6 0 2.912576 -8.126374 -6.251599

204 6 0 2.900191 -7.978891 -1.177120

205 6 0 2.932334 -8.756459 -8.635688

206 6 0 1.500790 -8.234091 1.203625

207 6 0 1.491846 -8.174041 -6.276082

208 6 0 1.500790 -8.234091 -1.203625

209 6 0 1.514794 -8.793981 -8.628608

210 6 0 0.756099 -8.328942 0.000000

211 6 0 0.760203 -8.497567 -7.454217

212 6 0 -0.756099 -8.328942 0.000000

213 6 0 -0.760203 -8.497567 -7.454217

214 6 0 -1.500790 -8.234091 1.203625

215 6 0 -1.491846 -8.174041 -6.276082

216 6 0 -1.500790 -8.234091 -1.203625

217 6 0 -1.514794 -8.793981 -8.628608

218 6 0 -2.900191 -7.978891 1.177120

219 6 0 -2.912576 -8.126374 -6.251599

220 6 0 -2.900191 -7.978891 -1.177120

221 6 0 -2.932334 -8.756459 -8.635688

222 6 0 -3.605779 -7.697625 2.491514

223 6 0 -3.608122 -7.732127 -4.963346

224 6 0 -3.626189 -8.413664 -7.446112

225 6 0 -4.727015 -6.827552 2.513774

226 6 0 -4.724810 -6.851502 -4.957174

227 6 0 -6.827552 -4.727015 2.513774

228 6 0 -6.851502 -4.724810 -4.957174

229 6 0 -7.697625 -3.605779 2.491514

230 6 0 -7.732127 -3.608122 -4.963346

231 6 0 -8.413664 -3.626189 -7.446112

232 6 0 -7.978891 -2.900191 1.177120

233 6 0 -8.126374 -2.912576 -6.251599

234 6 0 -7.978891 -2.900191 -1.177120

235 6 0 -8.756459 -2.932334 -8.635688

236 6 0 -8.234091 -1.500790 1.203625

237 6 0 -8.174041 -1.491846 -6.276082

238 6 0 -8.234091 -1.500790 -1.203625

239 6 0 -8.793981 -1.514794 -8.628608

240 6 0 -8.328942 -0.756099 0.000000

241 6 0 -8.497567 -0.760203 -7.454217

242 6 0 -8.328942 0.756099 0.000000

243 6 0 -8.497567 0.760203 -7.454217

244 6 0 -8.234091 1.500790 1.203625

245 6 0 -8.174041 1.491846 -6.276082

246 6 0 -8.234091 1.500790 -1.203625

247 6 0 -8.793981 1.514794 -8.628608

248 6 0 -7.978891 2.900191 1.177120

249 6 0 -8.126374 2.912576 -6.251599

250 6 0 -7.978891 2.900191 -1.177120

251 6 0 -8.756459 2.932334 -8.635688

252 6 0 -7.697625 3.605779 2.491514

253 6 0 -7.732127 3.608122 -4.963346

254 6 0 -8.413664 3.626189 -7.446112

255 6 0 -6.827552 4.727015 2.513774

256 6 0 -6.851502 4.724810 -4.957174

257 1 0 0.986313 -8.267032 2.175440

258 1 0 0.952317 -7.916588 -5.351699

259 1 0 0.986313 -8.267032 -2.175440

260 1 0 0.994780 -9.051416 -9.566255

261 1 0 -0.986313 -8.267032 -2.175440

262 1 0 -0.994780 -9.051416 -9.566255

263 1 0 -0.986313 -8.267032 2.175440

264 1 0 -0.952317 -7.916588 -5.351699

265 1 0 2.211369 -8.853707 3.724587

266 1 0 2.211369 -8.853707 -3.724587

267 1 0 -2.211369 -8.853707 3.724587

268 1 0 -2.211369 -8.853707 -3.724587

269 1 0 -4.729850 -8.382767 -7.446781

270 1 0 -5.104738 -6.476478 1.539010

271 1 0 -5.110828 -6.493237 -5.925597

272 1 0 -6.476478 -5.104738 1.539010

273 1 0 -6.493237 -5.110828 -5.925597

274 1 0 -8.382767 -4.729850 -7.446781

275 1 0 -8.267032 -0.986313 2.175440

276 1 0 -7.916588 -0.952317 -5.351699

277 1 0 -6.493237 -5.110828 5.925597

278 1 0 -6.476478 -5.104738 -1.539010

279 1 0 -5.110828 -6.493237 5.925597

280 1 0 -5.104738 -6.476478 -1.539010

281 1 0 -8.853707 -2.211369 3.724587

282 1 0 -8.853707 -2.211369 -3.724587

283 1 0 -8.267032 0.986313 2.175440

284 1 0 -7.916588 0.952317 -5.351699

285 1 0 -8.267032 -0.986313 -2.175440

286 1 0 -9.051416 -0.994780 -9.566255

287 1 0 -8.267032 0.986313 -2.175440

288 1 0 -9.051416 0.994780 -9.566255

289 1 0 -8.853707 2.211369 3.724587

290 1 0 -8.853707 2.211369 -3.724587

291 1 0 -8.382767 4.729850 -7.446781

292 1 0 -6.476478 5.104738 1.539010

293 1 0 -6.493237 5.110828 -5.925597

294 1 0 -5.104738 6.476478 1.539010

295 1 0 -5.110828 6.493237 -5.925597

296 1 0 -5.110828 6.493237 5.925597

297 1 0 -5.104738 6.476478 -1.539010

298 1 0 -6.493237 5.110828 5.925597

299 1 0 -6.476478 5.104738 -1.539010

300 1 0 -2.211369 8.853707 3.724587

301 1 0 -2.211369 8.853707 -3.724587

302 1 0 -4.729850 8.382767 -7.446781

303 1 0 -0.986313 8.267032 -2.175440

304 1 0 -0.994780 9.051416 -9.566255

305 1 0 -0.986313 8.267032 2.175440

306 1 0 -0.952317 7.916588 -5.351699

307 1 0 0.986313 8.267032 2.175440

308 1 0 0.952317 7.916588 -5.351699

309 1 0 0.986313 8.267032 -2.175440

310 1 0 0.994780 9.051416 -9.566255

311 1 0 4.729850 8.382767 -7.446781

312 1 0 2.211369 8.853707 3.724587

313 1 0 2.211369 8.853707 -3.724587

314 1 0 5.104738 6.476478 1.539010

315 1 0 5.110828 6.493237 -5.925597

316 1 0 5.110828 6.493237 5.925597

317 1 0 5.104738 6.476478 -1.539010

318 1 0 6.493237 5.110828 5.925597

319 1 0 6.476478 5.104738 -1.539010

320 1 0 6.476478 5.104738 1.539010

321 1 0 6.493237 5.110828 -5.925597

322 1 0 8.853707 2.211369 3.724587

323 1 0 8.853707 2.211369 -3.724587

324 1 0 8.382767 4.729850 -7.446781

325 1 0 8.267032 0.986313 2.175440

326 1 0 7.916588 0.952317 -5.351699

327 1 0 8.267032 0.986313 -2.175440

328 1 0 9.051416 0.994780 -9.566255

329 1 0 8.267032 -0.986313 -2.175440

330 1 0 9.051416 -0.994780 -9.566255

331 1 0 8.267032 -0.986313 2.175440

332 1 0 7.916588 -0.952317 -5.351699

333 1 0 8.382767 -4.729850 -7.446781

334 1 0 8.853707 -2.211369 3.724587

335 1 0 8.853707 -2.211369 -3.724587

336 1 0 6.476478 -5.104738 1.539010

337 1 0 6.493237 -5.110828 -5.925597

338 1 0 5.104738 -6.476478 1.539010

339 1 0 5.110828 -6.493237 -5.925597

340 1 0 5.110828 -6.493237 5.925597

341 1 0 5.104738 -6.476478 -1.539010

342 1 0 6.493237 -5.110828 5.925597

343 1 0 6.476478 -5.104738 -1.539010

344 1 0 4.729850 -8.382767 -7.446781

345 6 0 -9.106447 -3.712093 9.915189

346 1 0 -10.176695 -4.005364 9.911372

347 1 0 -8.503507 -4.636643 9.990147

348 1 0 -8.925768 -3.094288 10.814305

349 6 0 -3.712093 -9.106447 9.915189

350 1 0 -4.636643 -8.503507 9.990147

351 1 0 -4.005364 -10.176695 9.911372

352 1 0 -3.094288 -8.925768 10.814305

353 6 0 -9.106447 3.712093 9.915189

354 1 0 -8.925768 3.094288 10.814305

355 1 0 -8.503507 4.636643 9.990147

356 1 0 -10.176695 4.005364 9.911372

357 6 0 -3.712093 9.106447 9.915189

358 1 0 -4.005364 10.176695 9.911372

359 1 0 -4.636643 8.503507 9.990147

360 1 0 -3.094288 8.925768 10.814305

361 6 0 3.712093 9.106447 9.915189

362 1 0 3.094288 8.925768 10.814305

363 1 0 4.636643 8.503507 9.990147

364 1 0 4.005364 10.176695 9.911372

365 6 0 9.106447 3.712093 9.915189

366 1 0 10.176695 4.005364 9.911372

367 1 0 8.503507 4.636643 9.990147

368 1 0 8.925768 3.094288 10.814305

369 6 0 9.106447 -3.712093 9.915189

370 1 0 8.925768 -3.094288 10.814305

371 1 0 8.503507 -4.636643 9.990147

372 1 0 10.176695 -4.005364 9.911372

373 6 0 3.712093 -9.106447 9.915189

374 1 0 4.005364 -10.176695 9.911372

375 1 0 4.636643 -8.503507 9.990147

376 1 0 3.094288 -8.925768 10.814305

377 6 0 -3.712093 -9.106447 -9.915189

378 1 0 -4.005364 -10.176695 -9.911372

379 1 0 -4.636643 -8.503507 -9.990147

380 1 0 -3.094288 -8.925768 -10.814305

381 6 0 -9.106447 -3.712093 -9.915189

382 1 0 -8.503507 -4.636643 -9.990147

383 1 0 -10.176695 -4.005364 -9.911372

384 1 0 -8.925768 -3.094288 -10.814305

385 6 0 -9.106447 3.712093 -9.915189

386 1 0 -8.503507 4.636643 -9.990147

387 1 0 -8.925768 3.094288 -10.814305

388 1 0 -10.176695 4.005364 -9.911372

389 6 0 -3.712093 9.106447 -9.915189

390 1 0 -4.636643 8.503507 -9.990147

391 1 0 -4.005364 10.176695 -9.911372

392 1 0 -3.094288 8.925768 -10.814305

393 6 0 3.712093 9.106447 -9.915189

394 1 0 4.636643 8.503507 -9.990147

395 1 0 3.094288 8.925768 -10.814305

396 1 0 4.005364 10.176695 -9.911372

397 6 0 9.106447 3.712093 -9.915189

398 1 0 8.503507 4.636643 -9.990147

399 1 0 10.176695 4.005364 -9.911372

400 1 0 8.925768 3.094288 -10.814305

401 6 0 9.106447 -3.712093 -9.915189

402 1 0 8.503507 -4.636643 -9.990147

403 1 0 8.925768 -3.094288 -10.814305

404 1 0 10.176695 -4.005364 -9.911372

405 6 0 3.712093 -9.106447 -9.915189

406 1 0 4.636643 -8.503507 -9.990147

407 1 0 4.005364 -10.176695 -9.911372

408 1 0 3.094288 -8.925768 -10.814305

409 7 0 7.808477 3.657345 0.000000

410 7 0 3.657345 7.808477 0.000000

411 7 0 -3.657345 7.808477 0.000000

412 7 0 -7.808477 3.657345 0.000000

413 7 0 -7.808477 -3.657345 0.000000

414 7 0 -3.657345 -7.808477 0.000000

415 7 0 3.657345 -7.808477 0.000000

416 7 0 7.808477 -3.657345 0.000000

---------------------------------------------------------------------
