# Supplementary material for: A nitrogen-doped nanotube molecule with atom vacancy defects
Source: Nat Commun. 2020 Apr 14;11:1807. doi: 10.1038/s41467-020-15662-6 (PMC7156684; doi:10.1038/s41467-020-15662-6)
Supplement: Supplementary file 5 — Supplementary Data 2 [file 41467_2020_15662_MOESM5_ESM.doc]

**Supplementary Data 2 | Cartesian coordinates of 4,4'-bipyridine.**

---------------------------------------------------------------------

Center Atomic Atomic Coordinates (Angstroms)

Number Number Type X Y Z

---------------------------------------------------------------------

1 6 0 -2.914464 -1.130238 -0.272924

2 6 0 -1.502910 -1.178257 -0.281882

3 6 0 -0.754298 0.000002 -0.000001

4 6 0 -1.502911 1.178258 0.281882

5 6 0 -2.914467 1.130236 0.272921

6 1 0 -3.492366 -2.049372 -0.499901

7 1 0 -0.988345 -2.124182 -0.524365

8 1 0 -0.988350 2.124184 0.524368

9 1 0 -3.492369 2.049370 0.499898

10 6 0 0.754298 0.000002 0.000001

11 6 0 1.502911 1.178258 -0.281882

12 6 0 1.502910 -1.178257 0.281882

13 6 0 2.914467 1.130236 -0.272921

14 1 0 0.988350 2.124184 -0.524367

15 6 0 2.914464 -1.130238 0.272923

16 1 0 0.988345 -2.124182 0.524364

17 1 0 3.492369 2.049370 -0.499898

18 1 0 3.492366 -2.049372 0.499901

19 7 0 -3.696345 -0.000001 -0.000002

20 7 0 3.696345 -0.000001 0.000002

---------------------------------------------------------------------
